# Effect of Hybrid Type and Harvesting Season on Phytochemistry and Antibacterial Activity of Extracted Metabolites from *Salix* Bark

**DOI:** 10.1021/acs.jafc.1c08161

**Published:** 2022-02-24

**Authors:** Jinze Dou, Polina Ilina, Jarl Hemming, Kiia Malinen, Heidi Mäkkylä, Natália Oliveira de Farias, Päivi Tammela, Gisela de Aragão Umbuzeiro, Riikka Räisänen, Tapani Vuorinen

**Affiliations:** † Department of Bioproducts and Biosystems, School of Chemical Engineering, Aalto University, Espoo 02150, Finland; ‡ Drug Research Program, Division of Pharmaceutical Biosciences, Faculty of Pharmacy, University of Helsinki, Helsinki 00014, Finland; § Johan Gadolin Process Chemistry Centre, c/o Laboratory of Natural Materials Technology, Åbo Akademi University, Turku 20500, Finland; ∥ Laboratory of Ecotoxicology and Genotoxicity—LAEG, School of Technology, University of Campinas, Campinas 13083-970, Brazil; ⊥ HELSUS Helsinki Institute of Sustainability Science, Craft Studies, University of Helsinki, Helsinki 00014, Finland

**Keywords:** antimicrobial activity, mutagenicity, raffinose, toxicity, triandrin, water extract, willow bark

## Abstract

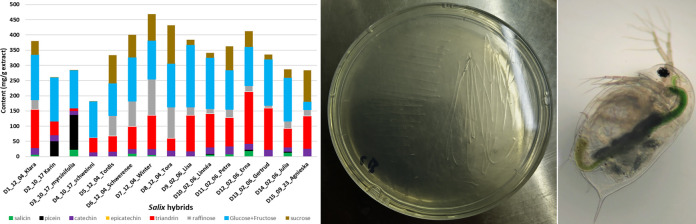

Hundreds of different fast-growing *Salix* hybrids have been developed mainly for energy crops. In this paper, we studied water extracts from the bark of 15 willow hybrids and species as potential antimicrobial additives. Treatment of ground bark in water under mild conditions extracted 12–25% of the dry material. Preparative high-performance liquid chromatography is proven here as a fast and highly efficient tool in the small-scale recovery of raffinose from *Salix* bark crude extracts for structural elucidation. Less than half of the dissolved material was assigned by chromatographic (gas chromatography and liquid chromatography) and spectroscopic (mass spectrometry and nuclear magnetic resonance spectroscopy) techniques for low-molecular-weight compounds, including mono- and oligosaccharides (sucrose, raffinose, and stachyose) and aromatic phytochemicals (triandrin, catechin, salicin, and picein). The composition of the extracts varied greatly depending on the hybrid or species and the harvesting season. This information generated new scientific knowledge on the variation in the content and composition of the extracts between *Salix* hybrids and harvesting season depending on the desired molecule. The extracts showed high antibacterial activity on *Staphylococcus aureus* with a minimal inhibitory concentration (MIC) of 0.6–0.8 mg/mL; however, no inhibition was observed against *Escherichia coli*, *Enterococcus faecalis,* and *Salmonella typhimurium*. MIC of triandrin (i.e., 1.25 mg/mL) is reported for the first time. Although antibacterial triandrin and (+)-catechin were present in extracts, clear correlation between the antibacterial effect and the chemical composition was not established, which indicates that antibacterial activity of the extracts mainly originates from some not yet elucidated substances. Aquatic toxicity and mutagenicity assessments showed the safe usage of *Salix* water extracts as possible antibacterial additives.

## Introduction

While the viral disease COVID-19 is sweeping the world, its lethality appears to be associated with the secondary bacterial sequelae, such as the ventilator-acquired bacterial pneumonia, as the lung has no defensive barrier against this bacterium.^1^ Such defenseless state is partially caused by the antimicrobial resistance (AMR) of the bacteria, developed, for example, by the antibiotics used in indoor poultry farming to promote growth and reduce chicken mortality. Although the mechanism of growth promotion in food animals is not fully established, antimicrobial agents are presumed to maintain intestinal health by regulating bacterial populations of the microbiome. Use of traditional antibiotics causes the resistance of pathogenic bacteria, which subsequently pose a health threat to human—for example, *Campylobacter* and *Salmonella* pathogens, both resistant to quinolones, can infect animals and humans.^2^

In 2006, EU banned the use of antibiotics as growth promoters in animal feed.^3^ Currently, only antimicrobial feed additives (e.g., ionophore coccidiostats) are allowed for their antimicrobial and antiparasitic properties. European AMR Surveillance Network (EARS-Net) reported that AMR infections have been responsible for 33 000 annual deaths by 2015.^4^ Nevertheless, the global need of the antimicrobials in food animals is predicted to rise by 67% from 63 151 tons (year 2010) to 105 596 tons (year 2030).^5^ Therefore, there has been a great interest in developing novel antibiotic alternatives to hinder the further growth of the AMR in animals and humans.

In the years 1981–2019, the naturally or mimic-based naturally occurring metabolites represented almost half of the newly registered drugs by FDA (U.S. Food and Drug Administration), thus their share being almost 2 times higher than that of the synthetic drugs (24.6%).^6^ Natural metabolites have similar beneficial effects as traditional antibiotics.^2^ The dietary willow (*Salix alba*) bark extract in the poultry feed has reduced significantly proliferation of pathogenic bacteria (i.e., *Enterobacteriaceae*) under heat stress and also stimulated growth of the favorable bacteria, such as lactobacilli, in the gut of broilers.^7^ Furthermore, the supply chain of other nature antimicrobials (e.g., essential oils, functional peptides, nisin, lysozymes, and so forth) may not as sustainable as willow tree because the renewable willow can be sustainably grown on abandoned peatland,^7^ which can help in soil carbon sequestration and thus promote carbon-neutral societies. Moreover, the pharmacological and stress-regulating activities of *Salix* phytochemicals are well known. Salicin has traditionally been made of willow bark, inhibiting the growth of both *Helicobacter pylori* by suppressing the mutagenic effect of metronidazole^8^ and *Staphylococcus aureus* (*S. aureus*) bacteria.^9^ Picein, the major metabolite in the willow bark water extracts,^10^ has a significant role in the defensing mechanism against spruce budworm.^11^ Triandrin is a stress regulator for the plant’s adaptogenic activity.^12^ Catechin is also a stress regulator that potentiates the antimicrobial activity of gentamicin against *S. aureus* and *Pseudomonas aeruginosa* (*P. aeruginosa*).^13^ Raffinose is a natural trisaccharide containing galactose, glucose, and fructose, which are prebiotic agents that promote the growth of lactobacilli.^14^

Although many studies on willow extractives have been published, a systematic approach for upgrading and utilizing these novel metabolites is still lacking, especially for the highly productive energy willow. It is hypothesized that pure extractive compounds can be produced in high yields through selection of the species or hybrid and harvesting season. The overall objective of this study was to evaluate the potential use of willow bark water extracts as antibiotic alternatives. We aimed to gain the knowledge on the variations in the water extract content, phytochemical composition, and their antibacterial activities. Furthermore, an in vivo toxicological and mutagenic evaluation of crude extracts was performed to assess their safe usages for animals and environment.

## Materials and Methods

### Materials and Chemicals

Fifteen willow hybrids (Tables S1 and S2) were harvested and supplied from Carbons Finland Oy, VTT Technical Research Center of Finland Ltd., and Lantmännen Lantbruk. The bark was manually peeled and cut into 2 cm pieces and stored at −20 °C for further use. The barks were ground into 1 mm particle size and dried at 40 °C in an oven. Acetone, arabinose, *N*,*O*-bistrifluoroacetamide (BSTFA), (+)-catechin, DMSO-*d*_6_, fructose, galactose, glucose, heneicosylic acid (C21 acid), mannose, picein, pyridine-*d*5, raffinose, rhamnose, salicin, trimethylsilyl chloride (TMCS), xylitol, and xylose were supplied from Sigma-Aldrich, Finland. Betulinol (99.5% purity) was supplied by Åbo Akademi University. Triandrin was purified by chromatography (Figure S1) based on its size and hydrophobicity differences among all bioactive molecules from willow hybrid Karin bark extract.^15^ The purity (ca. 90%) of triandrin was assessed from its nuclear magnetic resonance (NMR) spectra (Figures S2–S4).^16^ The exact match between the willow hybrid and their associated experiment is summarized in Table S1.

### Methods

#### Water Extraction

Water extraction was conducted in a silicon oil-bath-heated iron container by varying temperature (50–100 °C) and time (10–120 min) and using powdered willow bark at a liquid-to-solid ratio of 10:1. The water-soluble fraction was filtered using qualitative filter paper (particle retention 12–15 μm), centrifuged (8000 rpm) to remove any finer particles, and lyophilized. The solid water extract was stored covered with aluminum foil for further chemical analyses. Another set of 15 *Salix* hybrids (Tables S1 and S2) was extracted with water at 80 °C for 20 min. The extracts were purified from solid particles and lyophilized in the same manner as willow hybrid Klara.

#### Preparative High-Performance Liquid Chromatography for the Recovery of Raffinose

Preparative chromatography (Figure S5) was performed using Shimadzu preparative LC consisting of two LC-20AP pumps, degasser, SIL-10AP autosampler, SPD-M20A diode array detector, and FRC-10A fraction collector. The semipreparative Luna Omega 5 μm PS C18 100 Å (250 × 10 mm) column and Kinetex 5 μm Biphenyl 100 Å (250 × 10 mm) were used as a coupled column system for the separation. The bark extract of willow hybrid Tora was dissolved in water–acetonitrile mixture (92:8) using a concentration of 5 mg/mL. Ultrapure water–acetonitrile mixture (9:1) was used as an eluent with a flow rate of 3 mL/min. A desired fraction of interest (raffinose) was collected from 15 injections (200 μL/injection) with a retention time of 6.90–7.50 min (Figure S5) using a detection wavelength of 210 nm.

#### Antibacterial Activity

Four bacterial species reported as important bird pathogenic bacteria and zoonotic pathogens^17^ were selected as the test strains for this study. The strains used were *Enterococcus faecalis* ATCC 29213 and *S. aureus* ATCC 29213 (Gram-positive) and *Salmonella typhimurium* ATCC 19585 and *Escherichia coli* ATCC 25922 (Gram-negative). Evaluation of the antimicrobial activity of both the bark extracts and authentic pure metabolites was performed by broth microdilution assay following recommendations outlined by the CLSI standard.^18^ In brief, bacteria were grown on Mueller Hinton agar for 24 h at 37 °C and resuspended in sterile normal saline solution and turbidity of the bacterial suspension was evaluated using a McFarland densitometer (DEN-1, BioSan, Warren MI, USA). All willow bark extracts were dissolved in water at a concentration of 20 or 100 mg/mL. Picein, salicin, and triandrin were dissolved in water at a concentration of 20 mg/mL, (+)-catechin was dissolved in ethanol at 50 mg/mL, and (−)-epicatechin in 1:1 water/acetone at 10 mg/mL. The test samples were diluted in cation-adjusted Mueller Hinton broth to 2 times the final concentration. Minimum and maximum controls with the diluent and a positive control [ciprofloxacin at minimum inhibitory concentration (MIC)] were also prepared. 100 μL of the sample and control dilutions were transferred onto a clear sterile 96-well plate in triplicate, followed by 100 μL of the bacterial suspension of 10^6^ cfu/mL. No bacterial suspension was added into the minimum control. The assay plate was incubated at 37 °C with a shaking speed of 500 rpm (PST-60HL-4 Thermoshaker, BioSan), and bacterial growth was visually assessed after 24 h. The initial screening was performed at a concentration of 1 mg/mL (for bark extracts) or 3 mg/mL (for authentic metabolites). The selection of initial screening concentration was dictated by substance solubility and the amount of substance available for the analysis. Dose–response assays were performed for compounds demonstrating strong antibacterial activity, and MICs were determined as the lowest concentration resulting in no visible bacterial growth. We used the following dilution scheme: from 1 to 0.3 mg/mL with the dilution step 0.1 mg/mL for crude extracts and from 2 to 0.25 mg/mL with the dilution step 0.25 for (+)-catechin and triandrin. The dose–response assays were repeated two times. In cases when the obtained MIC values did not match but fell within ±1 dilution, we performed a third repeat and reported the MIC value obtained in two matching experiments out of three. Statistical analysis for the antibacterial activity study is not applicable to the data sets of two independent experiments (performed in triplicate wells).

#### Toxicological Evaluation

Acute aquatic toxicity of the willow (hybrid *Karin*) bark extract was evaluated with the freshwater microcrustacean *Daphnia similis* according to ABNT NBR 12713 and OECD 202.^19^ Solutions were prepared by dissolving lyophilized bark extract in dimethyl sulfoxide (DMSO). Test solutions were prepared in culture media using a maximum concentration of 0.1% of DMSO. A negative control was included in the test (0.1% DMSO). Twenty neonates (<24 h old) from 2- to 3-week old mothers were placed in four replicates for each concentration (five organisms/replicate). Tests were performed at 21 ± 1 °C under a photoperiod of 16 h light and 8 h darkness. After 48 h, the number of immobile daphnids was recorded. Tests were considered valid when immobility in the negative controls did not exceed 10%.

The mutagenicity was evaluated using the *Salmonella*/microsome mutagenicity assay at the limit of solubility in DMSO. Microplate Agar, a miniaturized protocol,^20^ was used to evaluate the mutagenic activity of two *Salmonella enterica* serovar *Typhimurium* strains (TA98 and TA100). Tests were performed in the absence and presence of the metabolic activation system provided by Aroclor 1254-induced Sprague Dawley rat liver S9 mix (MolTox, Boone, NC) with the required cofactors (S9 mixture) at 5% (v/v) concentration. DMSO was used as the negative control. Positive controls for TA98 and TA100 were 1.25 ng/μL nitroquinoline-oxide (4NQO) (Sigma-Aldrich) without S9 and 5 ng/μL 2-aminoanthracene (2AA) (Sigma-Aldrich) with S9. After 66 h incubation period, the number of revertant colonies was counted using a stereomicroscope. Toxicity was evaluated by the careful inspection of the background of the plates.

#### Statistical Analysis

For the toxicological studies, in the Daphnia acute toxicity test, data were statistically analyzed using the Hill 1 by the logistic regression method (Origin lab) for estimating the 50% effective (immobilization) concentrations (EC_50_). In the mutagenicity assay, experimental data were analyzed with the Salanal program (Integrated Laboratory Systems, Research Triangle Park, NC) using analysis of variance (ANOVA), followed by a linear regression. The sample was considered positive when both the ANOVA (*p* ≤ 0.05) and linear regression provided significant responses (*p* ≤ 0.05).

#### Chemical Characterization

##### Gas Chromatography

Roughly, 1 mg of the lyophilized crude water extract was trimethysilylated with a mixture of pyridine, BSTFA, and TMCS (1:4:1, v/v/v) at 70 °C for ca. 1 h. Xylitol, heneicosylic acid, and betulinol were used as internal standards to quantify the compounds of interest from the water extracts. The trimethylsilylated analytes were separated on a HP-I column (25 m × 0.2 mm, 0.11 μm film) and detected by flame ionization detection (FID). A temperature of 250 °C and a split ratio of 1:30 were set for the injector. Column oven temperature [gas chromatography (GC) I, Figure S6] was maintained at 100 °C for 8 min, increased first to 170 °C at 2 °C/min, and then to 310 °C at 12 °C/min. Another temperature program (GC II, Figure S7) was applied according to a previous study.^15^ The two different temperature programs (GC I and GC II) were designed for optimal separation and quantitation of the analytes in each case. “Mean” and “standard deviation” have been applied to calculate the average of the chemical composition of the *Salix* water extracts based on triplicate-independent measurements per each sample. Statistical analysis is not applied here. Standard deviations are shown as error bars of the mean, which indicates the variance of the reported results.

##### Quantitation of Monosaccharides by HPAEC-PAD

High-performance anion-exchange chromatography with pulsed amperometric detection (HPAEC-PAD) was applied as an alternative method to quantify monosaccharides in the bark extracts as described earlier.^10^

##### NMR Spectroscopy

^1^H, ^13^C, and ^1^H–^13^C heteronuclear single-quantum coherence (HSQC) measurements were conducted using a 400 MHz Bruker AVANCE III spectrometer. The samples were dissolved in DMSO-*d*_6_:pyridine-*d*5 (4:1).^21^ HSQC spectra were acquired (spectral widths of 10.5 and 165 ppm for ^1^H and ^13^C, respectively) using a relaxation delay (d1) of 2 s, 1K data points, 128 t1 increments, and 100 transients. ^1^H NMR spectra were acquired using a spectral width of 16 ppm, a d1 of 1s, and 64K data points. 1,3,5-Trioxane (δC 93.1, δH 5.12 ppm) was applied as an internal standard for the quantification of aromatic metabolites by ^1^H NMR spectroscopy. The following parameters were applied for ^13^C NMR spectroscopy: a spectral width of 236 ppm, a d1 of 2s, and 65K transients of 64K data points. The spectral images were processed using Topspin 4.0 (Bruker).

##### High-Resolution Mass Spectrometry

The high-resolution mass spectrometry (HRMS) spectra were acquired using an Agilent 6350 QTOF mass spectrometer (Santa Clara, USA) equipped with dual electrospray ionization (ESI) coupled to the Agilent 1260 Infinity HPLC system (Singapore). The flow rate was 0.25 mL/min (acetonitrile/ultrapure water, 1:1), and the injection volume was 2 μL. Analytes were measured in the positive ion mode, and the capillary voltage was set to +3500 V. Drying gas flow was set to 11 L/min with a temperature of 300 °C. Nebulizer, fragmentor, skimmer, and octopole radiofrequency were set to 25 psi, 150, 65, and 500 V, respectively. Mass measurement was performed in the HRMS mode with a mass range *m*/*z* 100–1100. The mass accuracy of the instrument using external calibration was specified to be ≤3 ppm.

## Results and Discussion

### Composition of Willow Bark Extracts

Previously, it was found that both temperature and time affected the extraction yield of willow (hybrid Karin) bark to some extent.^10^ The major components of the extract were glucose, fructose, triandrin, picein, and catechin, but the effect of temperature and time on the yield of individual compounds was not determined. Therefore, this aspect is further studied here to assist in the selection of the optimal analytical method for quantification. However, the systematic quantification of the individual compound under the effect of temperature and time is out of scope of this present study. This time, extraction was applied to ground bark (1 mm size), which led to ca. 5 wt % units higher gravimetric yield compared to bark cut to 2 cm length (Table S3). Obviously, the grinding of bark broke at least part of the cells and shortened the diffusion time dramatically. In further experiments, the extractions were still carried out at 80 °C for 20 min because the significant crude water extract yield can be achieved based on a study for willow hybrid Karin,^10^ although these phytochemicals for willow hybrid Klara can be extracted in water with ease at 50 °C within 10 min (Table S4).

To be sure about the reliability of the data, several analytical techniques were compared for the quantification of the phytochemicals in the extracts, although each measurement per analytic was performed once. The techniques applied included GC with two different column oven temperature programs, HPAEC–PAD and ^1^H NMR spectroscopy. Slower initial increase in the column oven temperature (GC-I) showed a better separation of individual monosaccharides by GC (Figures S6 and S7), although the temperature ramp-up rate did not really affect the quantitation of the total monosaccharide content (Tables S4 and S5). In comparison with GC, HPAEC-PAD tended to provide 14–61% higher glucose contents and 2–26% lower fructose contents (Table S6), but the difference in the analyzed overall monosaccharide content was 3.3–10.2% between the methods (Tables S4 and S5). The slower oven temperature increase (GC-II) in the end of the GC analysis provided slightly better separation (Figures S6 and S7) and higher response of the aromatic phytochemicals (Tables S4 and S5). Results from the quantitation of triandrin by two independent methods, GC-II and ^1^H NMR spectroscopy, correlated well, although the latter one provided 5.0–13.9% higher contents for all hybrids except the hybrid Klara (Figure S8). Based on all observations, GC-II was selected as an accurate and simple technique for the simultaneous quantitation of the aromatic compounds and sugars in the extracts.

**Figure 1:**
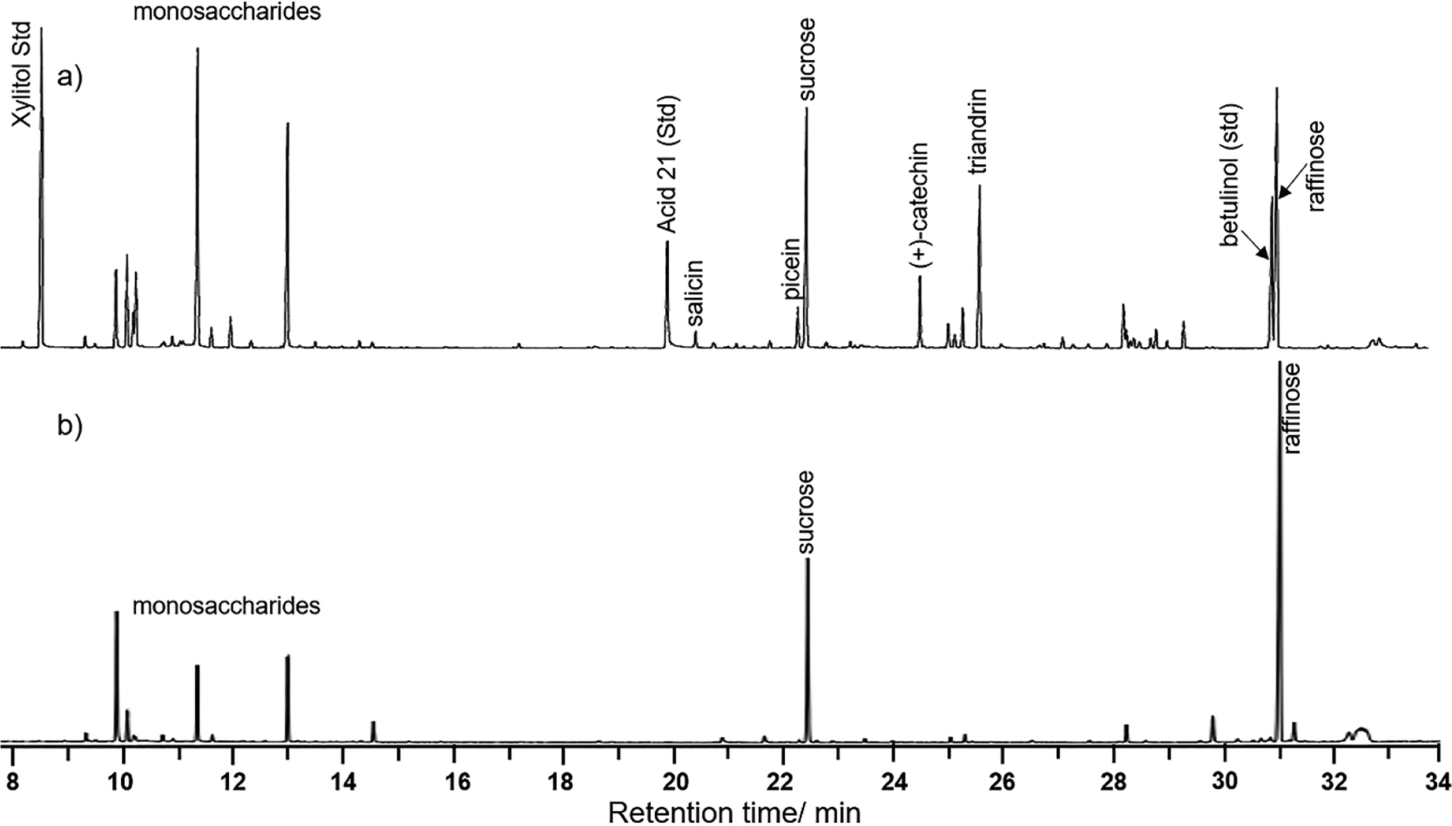
Gas chromatograms of the trimethylsilyl derivatives of the (a) bark extract of willow hybrid Tora (harvested on Dec 4) with the internal standard (Xylitol, Std) and (b) “raffinose-rich” fraction of the extract (Figure S5).

Knowledge on the variation in the entire chemical profile of the *Salix* bark extracts is necessary in understanding the origin of their potential antibacterial activity. In addition to the previously reported glucose, fructose, picein, triandrin, salicin, and (+)-catechin,^21−23^ sucrose and trace amounts of several neutral monosaccharides (Table S6) and glucuronic acid (Tables S4 and S5) were present in the extracts. Gas chromatograms of several extracts had an additional major peak close to betulinol that was used as an internal standard (Figures 1 and S9). The EI mass spectrum of this compound showed great similarity with the spectrum of trimethylsilylated sucrose (Figure S10). Match with the retention time of authentic raffinose supported the assignment to the trisaccharide that has been reported to be present in willow honey (Figure 1).^24^

**Figure 2:**
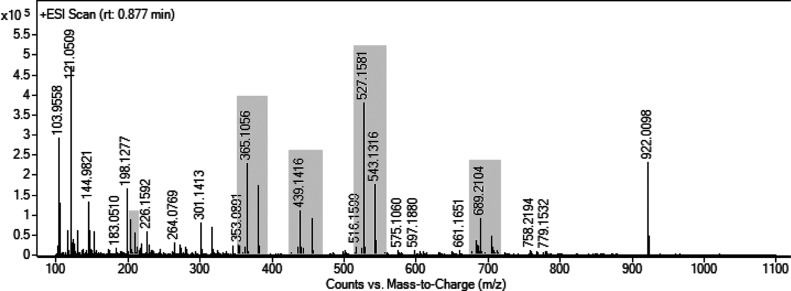
ESI HRMS spectrum (rt: 0.877 min) of the isolated “raffinose-rich” fraction (Figure S5).

**Figure 3:**
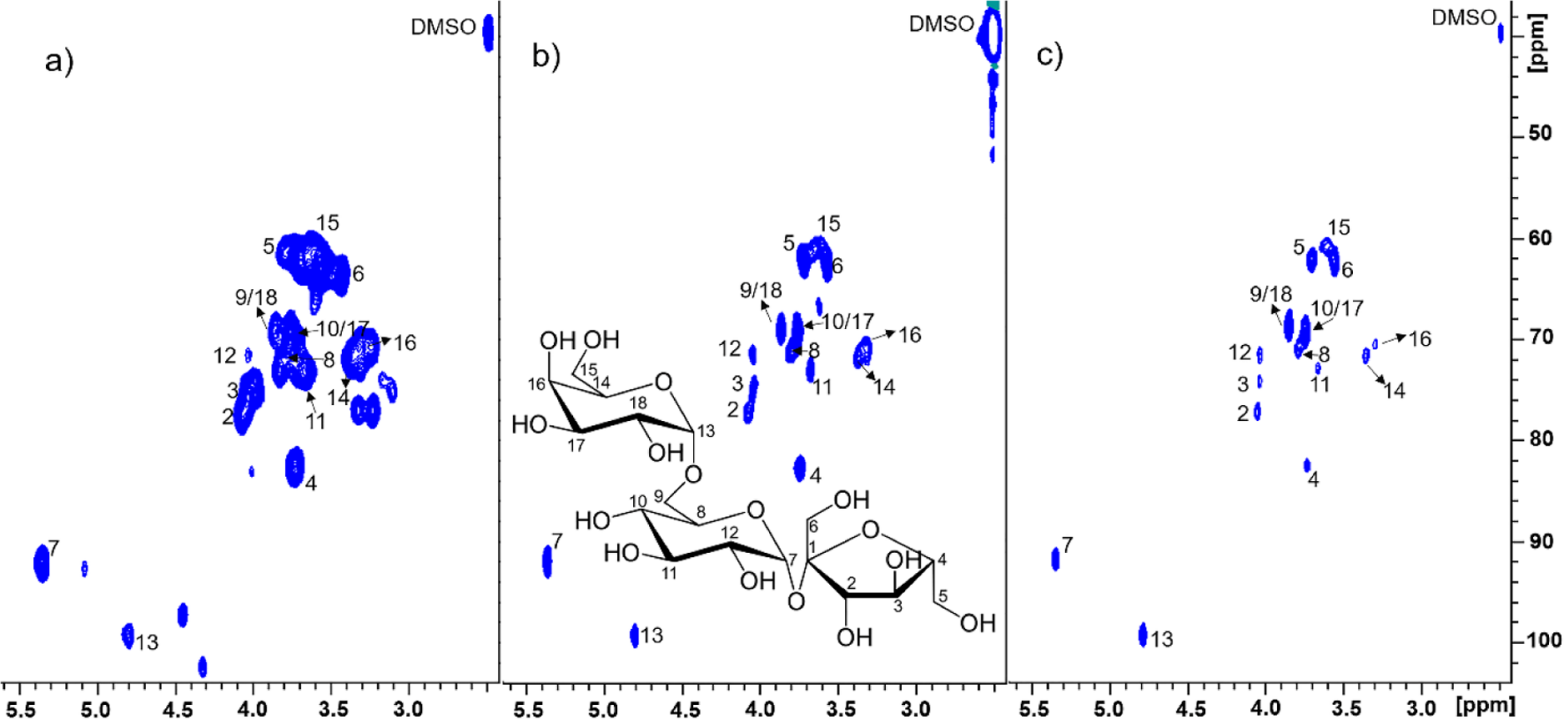
Carbohydrate region of 2D HSQC NMR spectra of the (a) bark extract of hybrid Tora (harvested on Dec 4) (Table S1); (b) “raffinose-rich” fraction of the extract (Figure S5); and (c) authentic raffinose in DMSO-*d*_6_/pyridine-*d*5 (4:1).

To confirm the assignment, preparative HPLC was applied to isolate a “raffinose-rich” fraction from the bark extract of willow hybrid Tora. The elemental composition determined by HRMS confirmed the presence of mono- (glucose and fructose), di- (sucrose), tri- (C_18_H_32_O_16_), and tetrasaccharides (C_24_H_42_O_21_) of which tri- and disaccharides were the most dominant ones in this order (Figure 2 and Table 1). The elemental composition of an additional component (C_15_H_28_O_13_) signaled the presence of a glycerol disaccharide, such as the rare sugar fuzinoside.^25^ A comparison between the NMR spectra of the original extract, its “raffinose-rich” fraction, and authentic raffinose confirmed its presence (Figures 3, S11, and S12).^26^ However, the HSQC NMR spectrum of the structurally related tetrasaccharide stachyose is very similar to the spectrum of raffinose which might hide the weaker stachyose signals.^26^ However, based on HRMS, a tetrasaccharide was present, which is here tentatively assigned as stachyose.

**Table 1:** Signal Assignments of Figure 2^a^

calculated *m*/*z*	found *m*/*z*	assignment
121.0509	121.0509	purine, reference mass correction
203.0526, 219.0265	203.0529, 219.0266	monosaccharide: [C_6_H_12_O_6_] + Na, [C_6_H_12_O_6_] + K
365.1054, 381.0794	365.1061, 381.0798	disaccharide: [C_12_H_22_O_11_] + Na, [C_12_H_22_O_11_] + K
417.1603, 439.1422, 455.1161	417.1602, 439.1424, 455.1163	disaccharide glycerol: [C_15_H_28_O_13_] + H, [C_15_H_28_O_13_] + Na, [C_15_H_28_O_13_] + K
505.1763, 527.1583, 543.1322	505.1769, 527.1589, 543.1325	trisaccharide: [C_18_H_32_O_16_] + H, [C_18_H_32_O_16_] + Na, [C_18_H_32_O_16_]+] + K
689.2111, 705.1850	689.2109, 705.1698	tetrasaccharide: [C_24_H_42_O_21_] + H, [C_24_H_42_O_21_] + Na, [C_24_H_42_O_21_] + K
922.0098	922.0098	hexakis(1*H*,1*H*,3*H*-tetrafluoropropoxy) phosphazene, reference mass correction

a The saccharides are formed of hexoses.

### Effect of Hybrid Type and Harvesting Season on Phytochemistry

**Figure 4:**
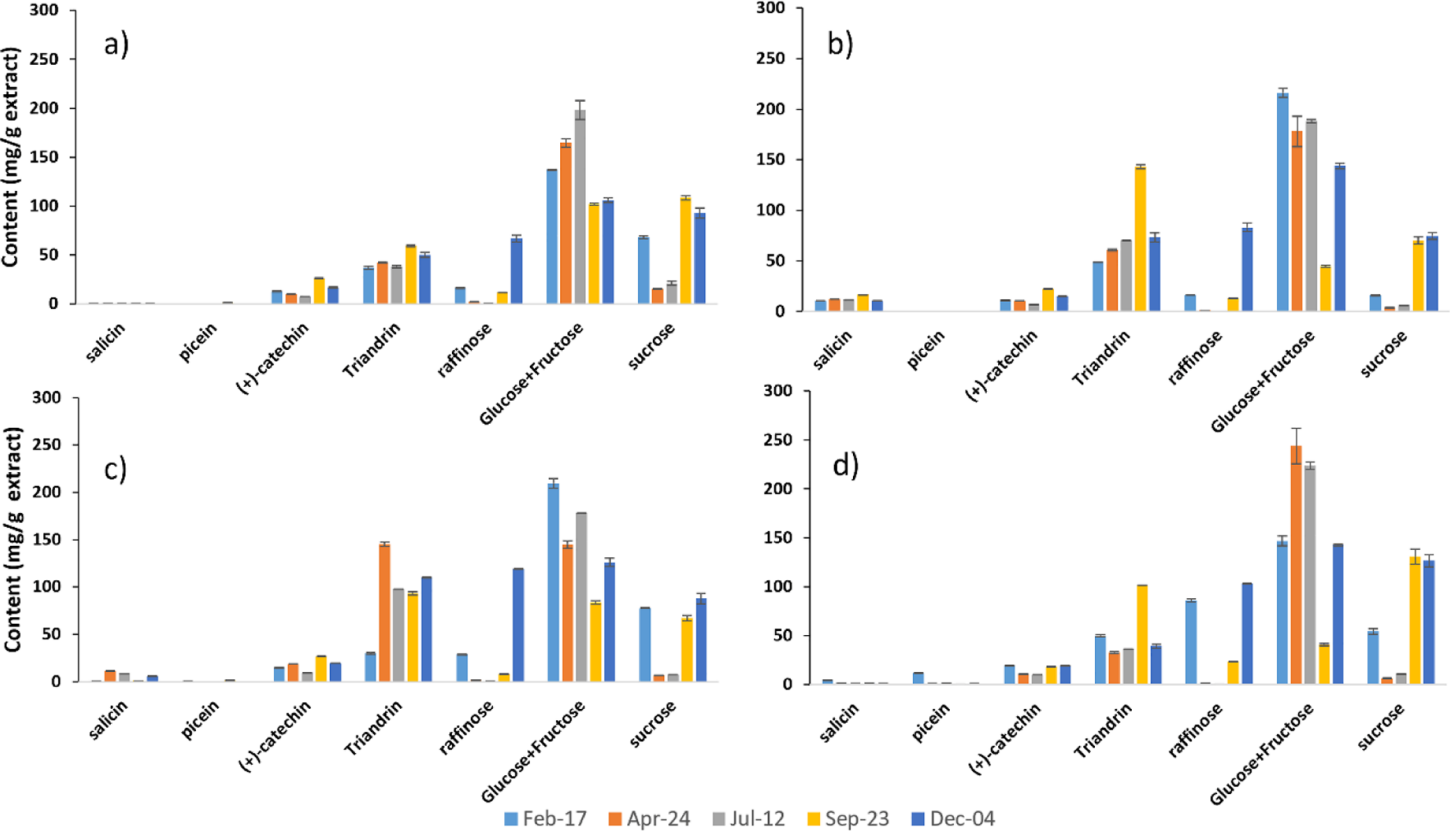
Chemical composition (mean = average of the content based on triplicate measurements) of water extracts quantified by GC-FID from selected *Salix* hybrids: (a) Tordis; (b) Schwerenee; (c) winter; and (d) Tora that were harvested in different seasons. Standard deviations (Table S7) are shown as error bars of the mean. The gravimetric extraction yields are summarized in Figure S13.

Both raffinose and stachyose are known as cryoprotectant oligosaccharides that have a high response to the cold hardening of several plants including *Salix*.^27^ In fact, the raffinose content of the bark extracts was the highest when the *Salix* stems were harvested during the winter (Figure 4). However, the seasonal effect on the content of aromatic components (i.e., salicin, picein, catechin, and triandrin) was not as clear as the effects on sucrose and raffinose in Figure 4.

**Figure 5:**
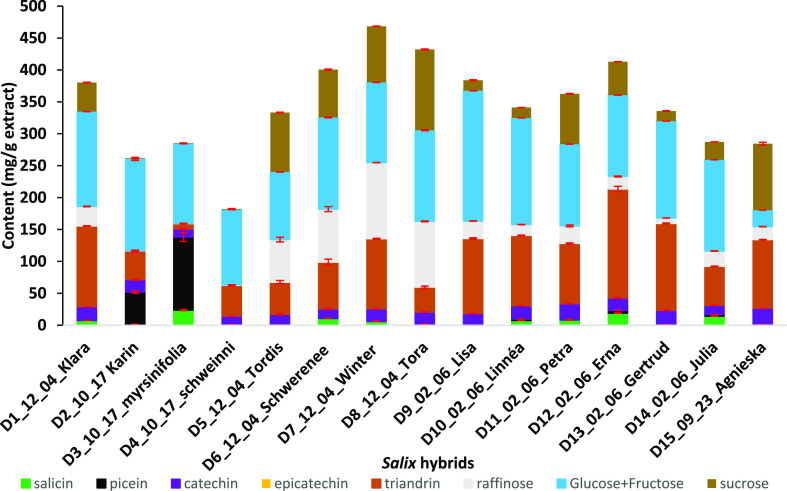
Chemical composition (mean = average of the content based on triplicate measurements) of water extracts from 15 *Salix* hybrids quantified by GC-FID. Sample abbreviations include hybrid code, harvesting month, harvesting day, and hybrid name, for example, D1_12_04_Klara refers to hybrid Klara harvested on December 4th. Standard deviations (Table S8) are shown as error bars (in red color) of the mean.

The aromatic phytochemical composition of the *Salix* bark extracts (Figure 5 and Table S8) varied largely depending on the hybrid or species. Only (+)-catechin was present approximately in an equal amount (15–25 mg/g) independent of the *Salix* hybrid. Catechin is quite unstable under neutral pH but rather stable under an acidic environment.^28^ Triandrin was always present, but its content showed large variation (8–170 mg/g) between the samples, whereas picein occurred only in *S. myrsinifolia* and hybrid Karin. In comparison with triandrin, the content of salicin was low and varied largely between the samples.

The gravimetric extraction yield varied between 12–25% depending on the sample (Figure S14), and the assigned phytochemicals corresponded to 18–47% of the extract (Table S8). Recently, preparative chromatography was applied for the efficient fractionation of willow hybrid Karin water extract.^15^ The unassigned fractions (ca. 50 wt %) included colored substances that could possibly originate from condensed tannins. In fact, ^13^C NMR spectra of the bark extracts contained several broad “background” signals at chemical shifts that are characteristic for polymeric condensed tannins (Figure S15).^29^ Condensed tannin (or proanthocyanidin) is a natural macromolecular polymer, with a molecular weight of 500–3000 Da, present at most vascular plants. It usually contains a group of flavonoids that derived mostly from flavan-3-ols repeating units and a smaller portion of saccharides. Flavonoids, polyphenol with C6–C3–C6 skeleton, are antibacterial agents against pathogenic microorganisms.^30,31^ Therefore, this biomass colorant (rich in flavonoids) may have the strongest antibacterial activity among the metabolites. However, this is beyond the scope of this present study.

### Antimicrobial Activity and Toxicological Evaluation

Hybrid selection of their bark extract is based on their different abundance (Table S9) of bioactive chemical components. Initial screening of bark extracts (1 mg/mL) of selected hybrids (Klara, Karin, *S. myrsinofolia*, Tora, and Erna) showed strong antibacterial activity against *S. aureus* but not against three other bacterial species *E. faecalis*, *S. typhimurium*, and *E. coli* (Table S9). *S. aureus* is a Gram-positive microorganism with widely occurring resistance to a variety of antibiotics, such as methicillin, and is in the global priority list of antibiotic-resistant bacteria by the World Health Organization.^32^ Activity against *S. aureus* was investigated in more detail by testing the activity of individual metabolites (triandrin, (+)-catechin, salicin, and picein) and determining MIC values for the compounds which inhibited bacterial growth, as well as MIC values of selected hybrid extracts. Only triandrin (MIC of 1.25 mg/mL) and (+)-catechin (MIC of 1.75 mg/mL)^33^ showed significant antibacterial activity against *S. aureus*, resulting in the eradication of viable bacteria after 24 h incubation (Table 2).

**Table 2:** MIC of Bark Extracts of Several *Salix* Hybrids, Organized in the Order of Their Ascending Triandrin Content and Authentic (+)-Catechin and Triandrin (Mean = Average of the Content Based on Triplicate Measurements) against *S. aureus* ATCC 29213 Based on Visual Assessment^a^

	sample	MIC mg/mL	triandrin (SD) mg/g	(+)-Catechin (SD)mg/g
bark extract	*S. myrsinofolia*	0.8	8 (0.5)	12 (0.7)
	Tora	0.7	39 (1.8)	19 (0.2)
	Karin	0.7	45 (1.3)	19 (0.6)
	Klara	0.8	127 (0.3)	21 (0)
	Erna	0.6	171 (4.1)	20 (0.5)
authentic compound	(+)-catechin	1.75		
	triandrin	1.25		

a SD = standard deviation of the mean.

One of the major mechanisms of AMR in bacteria is mediated by their ability to remove antibiotics from the cells. Thus, the resistant bacteria possess efficient efflux systems known as multidrug resistance (MDR) pumps, which could reduce the intracellular concentrations of antibiotics to an ineffective level. This also suggests that antibiotics can be more effective if the pump action can be inhibited.^34^ Although picein and salicin did not show any inhibition against *S. aureus* at concentrations up to 3 mg/mL, they may potentiate the discovered antimicrobials (triandrin and (+)-catechin) by inhibiting toward the NorA transporter (an MDR efflux pump) of *S. aureus*.^34^ This may explain the higher inhibition efficiency of the bark extract on the growth of *S. aureus* (MIC of 0.6–0.8 mg/mL),^35^ when compared to their individual metabolites. On the other hand, MIC values of the extracts did not correlate with their triandrin contents (Table 2). The MIC values of the (+)-catechin and the crude water extracts were comparable to those of previous studies;^33,35^ however, the MIC of triandrin was reported for the first time to the best of our knowledge.

These results show the diverse antimicrobial activity of *Salix* metabolites both from different hybrids and their individual phytochemicals. Even though some metabolites do not demonstrate antimicrobial activity, they may potentiate the known antimicrobial effect by inhibiting the microbial MDR pumps.

The concern for nontoxic alternative veterinary antimicrobial agents has been growing significantly as people are more aware of excessive carcinogenic effects to our environment from the commercial synthetic antibiotics (30–500 μg/L).^36^ Therefore, understanding the toxicological effect to the environment is a key step to utilize the willow bark water extracts as a source of an environmentally friendly antimicrobial agents.

Acute toxicity and mutagenicity of the willow bark water extracts were not observed under the tested conditions. No acute toxicity was observed for the microcrustacean *D. similis* at the limit of solubility (25 mg/L) because no statistical differences in the concentration response were observed when the logistic regression was applied (Table S10). Additionally, the willow bark water extracts did not present mutagenic activity in *S. typhimurium* TA98 and TA100 strains with and without S9 at 5% because no statistical increase in the number of revertant per well in comparison to the negative control was observed according to the ANOVA *p*-values (Table 3). Overall, our study has shown the promising potential of utilizing the willow bark water extracts in reducing the overwhelming dependence on reliance of the synthetic antibiotics.

**Table 3:** Mutagenic Evaluation of *Salix* Hybrid Karin Bark Water Extracts with the Strains TA98 and TA100 with and without Metabolic Activation (S9)^a^

		TA98		TA100	
		–S9	+S9	–S9	+S9
strain		number of revertant per well			
sample	concentration (ng/μL)	mean (SD) *N* = 4	mean (SD) *N* = 4	mean (SD) *N* = 4	mean (SD) *N* = 4
negative control (DMSO)	0	2 (1.15)	2 (1.41)	9.75 (1.5)	13 (4.55)
bark extract	37.5	1 (0.82)	1 (0.82)	6.5 (4.65)	Not tested
	75	2.25 (0.5)	2.75 (1.26)	10 (3.56)	11.25 (0.96)
	150	1.25 (0.5)	2 (0.82)	13.5 (1.91)	13.5 (2.65)
	300	1.75 (0.96)	0.75 (0.5)	9.25 (4.5)	10 (2.94)
	600	1.75 (0.5)	1 (0.82)	9.5 (2.65)	15 (1.41)
*p*-value for ANOVA^b^		0.231	0.103	0.258	0.152
positive control (4NQO/2AA)		53.75 (6.34)	150 (0)	150 (0)	150 (0)

a Mean = average of colonies in four wells/concentration; SD = standard deviation of the mean; −S9 = without metabolic activation; and +S9 = with metabolic activation.

b All ANOVA *p*-values are greater than 0.05.

In conclusion, the extract was nontoxic to Daphnia and nonmutagenic to bacteria and showed a high antibacterial effect on the growth of *S. aureus*. No direct correlation between the antibacterial activity and the chemical composition of the extracts was found. Raffinose is for the first time systematically purified through preparative HPLC, characterized through GC–MS/GC-FID examination, and further verified through GC-FID, NMR, and LC-HRMS. The bark extract composition varied greatly between the willow hybrids and species. Unlike triandrin and (+)-catechin, large amounts of picein were present in few cases only. The greatest seasonal variation was observed for sucrose and raffinose which were specifically present in samples harvested during winter. This exploration may launch a new era for applying *Salix* metabolites as genuinely novel antimicrobials to improve our resistance toward zoonotic pathogens, not only to enhance the growth productivity and gut health of food animals. Additional work will be needed to fully characterize the bark extracts (particularly the flavonoid-rich tannins) and the synergistic mechanisms behind their antibacterial activity. We will investigate, in vivo, the effect of *Salix* metabolites on animal performance and intestinal health. We aim to understand and establish the exact mechanisms associated with bacteriological changes in gut microbiota to optimize their use as effective antimicrobials, all of which are considered as a key step toward the complete willow biorefinery.^37,38^

## Supporting Information Available

Profile information, preparative-HPLC chromatogram, gas chromatogram, GC–MS spectrum, and NMR spectrum of the *Salix* hybrids (PDF)
