# Supplementary material for: Effect of Hybrid Type and Harvesting Season on Phytochemistry and Antibacterial Activity of Extracted Metabolites from Salix Bark
Source: J Agric Food Chem. 2022 Feb 24;70(9):2948–56. doi: 10.1021/acs.jafc.1c08161 (PMC8915259; doi:10.1021/acs.jafc.1c08161)
Supplement: Supplementary file 1 — jf1c08161_si_001.pdf [file jf1c08161_si_001.pdf]

The Effect of Hybrid Type and Harvesting Season on Phytochemistry and Antibacterial Activity of Extracted Metabolites from *Salix* bark

Jinze, Dou[1*]; Polina, Ilina[2]; Jarl, E. Hemming[3]; Kiia, Malinen[1]; Heidi, Mäkkylä[2]; Natália, Oliveira de Farias[4]; Päivi, Tammela[2]; Gisela, de Aragão Umbuzeiro[4]; Riikka, Räisänen[5]; Tapani, Vuorinen[1]

[1]Department of Bioproducts and Biosystems, School of Chemical Engineering, Aalto University, Finland

[2]Drug Research Program, Division of Pharmaceutical Biosciences, Faculty of Pharmacy, University of Helsinki, Finland

[3]Johan Gadolin Process Chemistry Centre, c/o Laboratory of Natural Materials Technology, Åbo Akademi University, Finland

[4]Laboratory of Ecotoxicology and Genotoxicity – LAEG, School of Technology, University of Campinas, Brazil

[5]HELSUS Helsinki Institute of Sustainability Science, Craft Studies, University of Helsinki, Finland

Table S1. Information on the *Salix* hybrids used in this study. Abbreviations: Kouvola (Aitomäki, Kouvola, Finland); Jyväskylä (Kyyjärvi, Jyväskylä, Finland); Svalöv (Svalöv, Sweden); Carbons (Carbons Finland Oy); VTT (VTT Technical Research Center of Finland Ltd.); Lantmännen (Lantmännen Lantbruk). Abbreviations for associating the willow hybrids with the applied experiments: 1) selection of hybrid Karin for Triandrin purification study by chromatographic **(C)** recovery and purification is because hybrid Karin contains equal presence of bioactive component including triandrin, picein and (+)-catechin; 2) The effect of extraction temperature/ time on the composition (mg/g) of water extract of bark **(E)**[1]; 3) The effect of hybrid type on the phytochemistry **(H)**; 4) The effect of harvesting season on the phytochemistry **(S)**[1]; 5) Selection of willow hybrid Tora for preparative-HPLC recovery of raffinose **(R)** is because hybrid Tora contains the highest amount of raffinose among the studied willow hybrids; 6) Selection of willow hybrids for the antibacterial activity **(A)** study is based on their different abundance of bioactive chemical components; 7) The selection of willow hybrid Karin for toxicological evaluation **(T)** is because hybrid Karin contains equal presence of bioactive component including triandrin, picein and (+)-catechin.

Abbreviation	Hybrid	Age	Growth location	Harvest time	Supplier	Applied experiment
D1_12_04_Klara	Klara	1	Kouvola	4th December, 2019	Carbons	E; H; A
D2_10_17_Karin	Karin	4	Jyväskylä	17th October, 2014	VTT	C; H; A; T
D3_10_17_myrsinifolia	myrsinifolia	4	Jyväskylä	17th October, 2014	VTT	H; A
D4_10_17_ schweinni	schweinni	4	Jyväskylä	17th October, 2014	VTT	H
D5_12_04_Tordis	Tordis	1	Kouvola	4th December, 2019	Carbons	H; S
D6_12_04_Schwerenee	Schwerenee	1	Kouvola	4th December, 2019	Carbons	H; S
D7_12_04_ Winter	Winter	1	Kouvola	4th December, 2019	Carbons	H; S
D8_12_04_ Tora	Tora	1	Kouvola	4th December, 2019	Carbons	H; S; R; A
D9_02_06_ Lisa	Lisa	1	Svalöv	6th February, 2020	Lantmännen	H
D10_02_06_Linnéa	Linnéa	1	Svalöv	7th February, 2020	Lantmännen	H
D11_02_06_Petra	Petra	1	Svalöv	8th February, 2020	Lantmännen	H
D12_02_06_Erna	Erna	1	Svalöv	9th February, 2020	Lantmännen	H; A
D13_02_06_Gertrud	Gertrud	1	Svalöv	10th February, 2020	Lantmännen	H
D14_02_06_Julia	Julia	1	Svalöv	11th February, 2020	Lantmännen	H
D15_09_23_Agnieska	Agnieska	1	Kouvola	23rd September, 2019	Carbons	H

[1]the hybrid selection for E and S is because of the high bioavailability of those hybrids for the planned study.

Table S2. Pedigree tree of *Salix* hybrids that were applied at this study.

Variety	Parents	Grandparents	Parents of grandparents
Karin	*Salix* hybrid	*Salix* Tora	*Salix* schwerinii Amgunskaja
			Salix viminalis (L 78195/ L78101)
		Salix viminalis	*Salix* viminalis L 820332
			Salix ciminalis L 81102
	Salix viminalis Ivar "Kirov"	"Kirov"	—
			—
		"Kirov"	—
			—
Klara	*Salix* hybrid	*Salix* hybrid	*Salix* hybrid SW 911310
			Salix viminalis Jorr
		Salix viminalis Ivar "Kirov"	—
			—
	Salix hybrid	*Salix* viminalis Bowles	—
			—
		Salix Bjorn	*Salix* Scwerinii Amgunskaja
			Salix Viminalis Orm
Lisa	*Salix* viminalis	—	—
	Salix schwerinii	—	—
Linnea	*Salix* viminalis	—	—
	Salix dacyclados	—	—
	Salix eriocephala	—	—
Petra	*Salix* viminalis	—	—
	Salix schwerinii	—	—
Erna	*Salix* viminalis	—	—
	Salix schwerinii	—	—
	Salix eriocephala	—	—
Gertrud	*Salix* viminalis	—	—
	Salix schwerinii	—	—
Julia	*Salix* viminalis	—	—
	Salix schwerinii	—	—
	Salix aurita	—	—

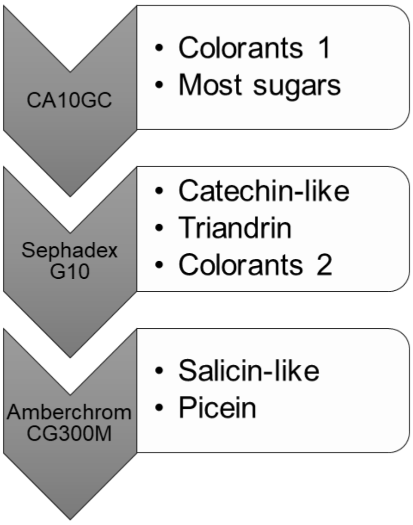

Figure S1. Chromatographic fractionation process of water extracts from willow (hybrid Karin) bark.[1]

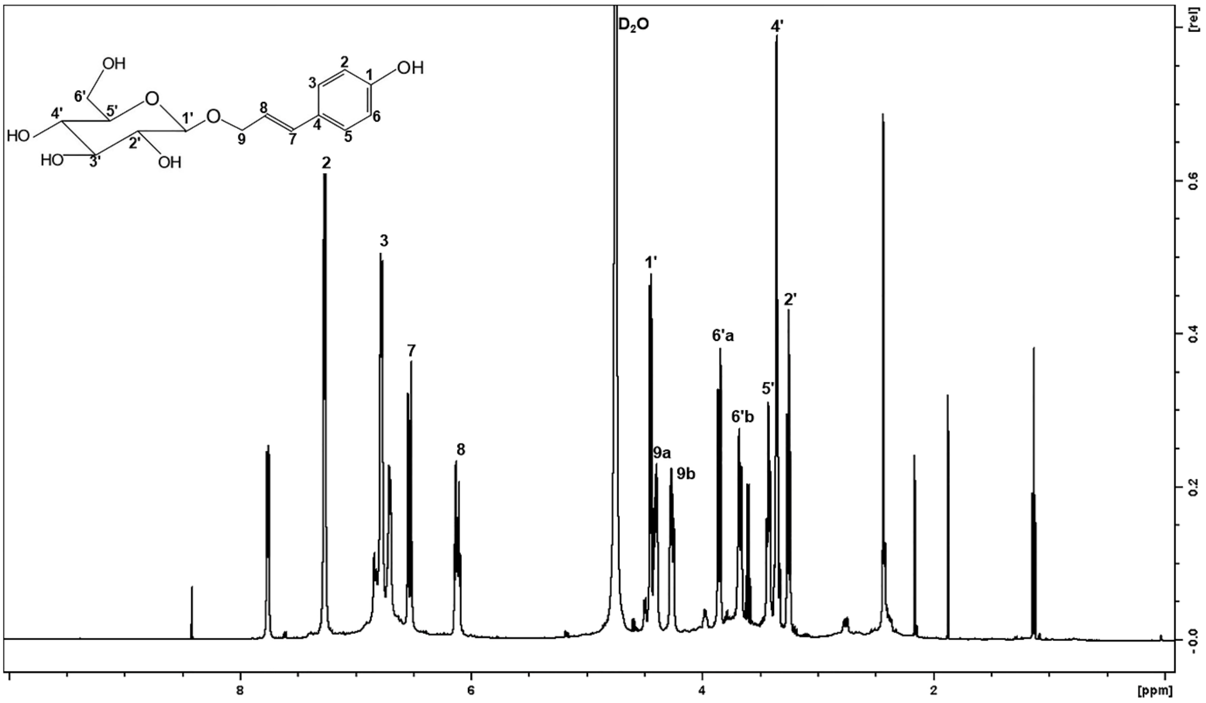

Figure S2. ^{1}H NMR spectrum of isolated triandrin (**Figure S1**) in D_2O.

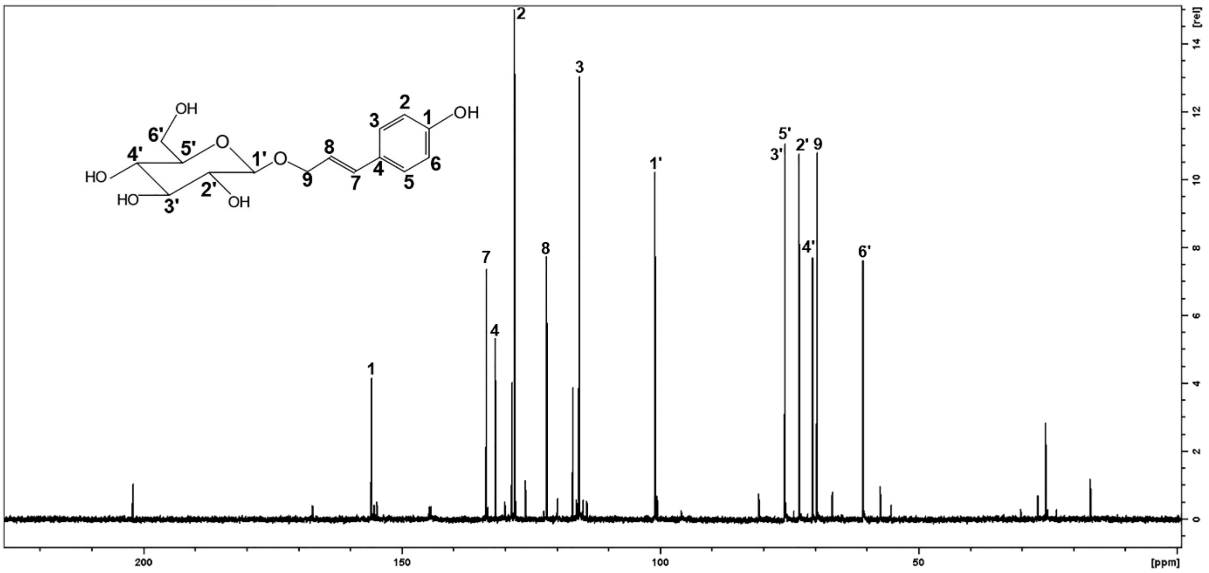

Figure S3. ^{13}C NMR spectrum of isolated triandrin (**Figure S1**) in D_2O.

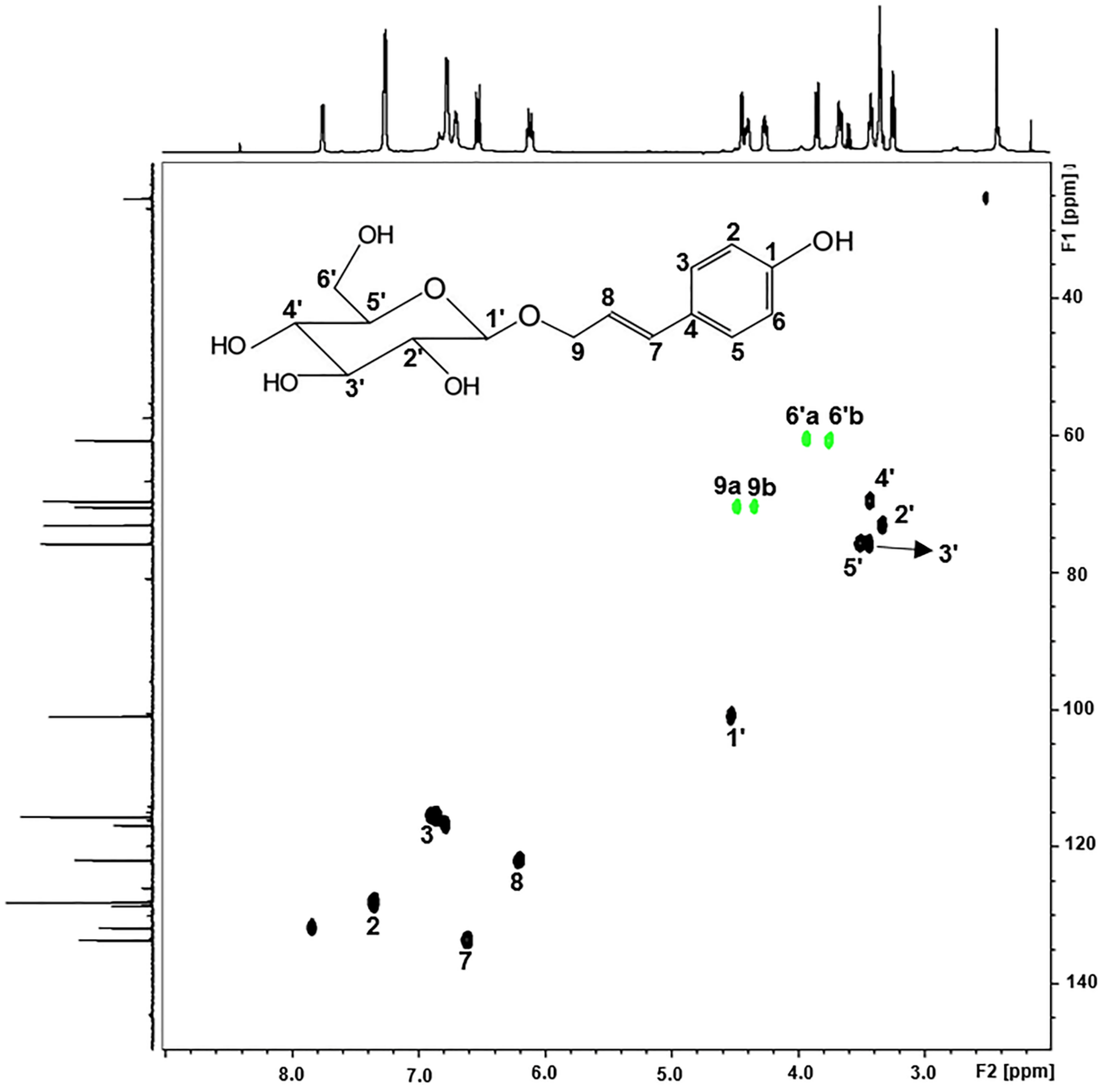

Figure S4. 2D ^{1}H–^{13}C HSQC NMR spectrum of isolated triandrin (**Figure S1**) in D_2O.

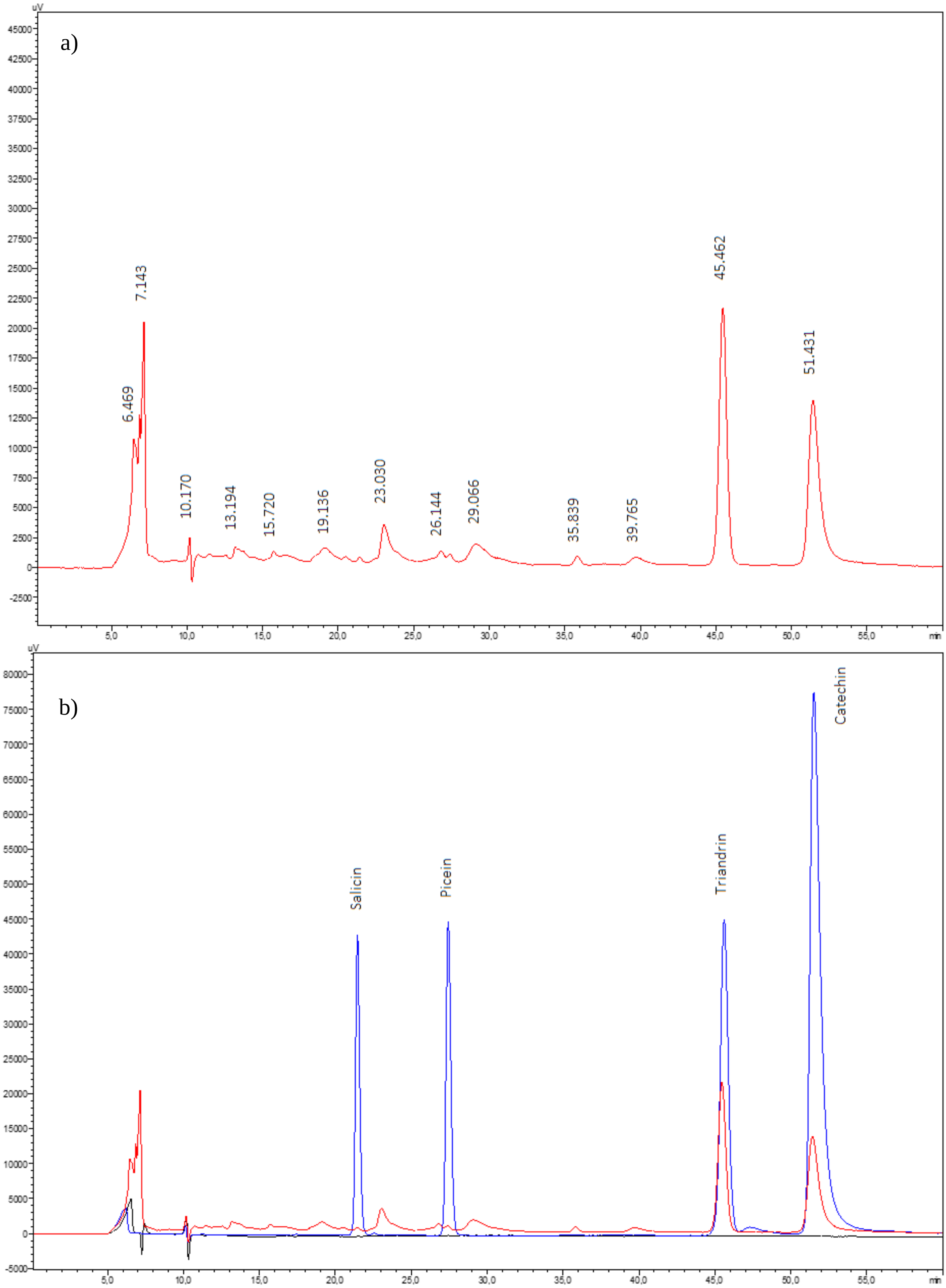
a)
uV
min
6.469
7.143
10.170
13.194
15.720
19.136
23.030
26.144
29.066
35.839
39.765
45.462
51.431
b)
Salicin
Picein
Triandrin
Catechin

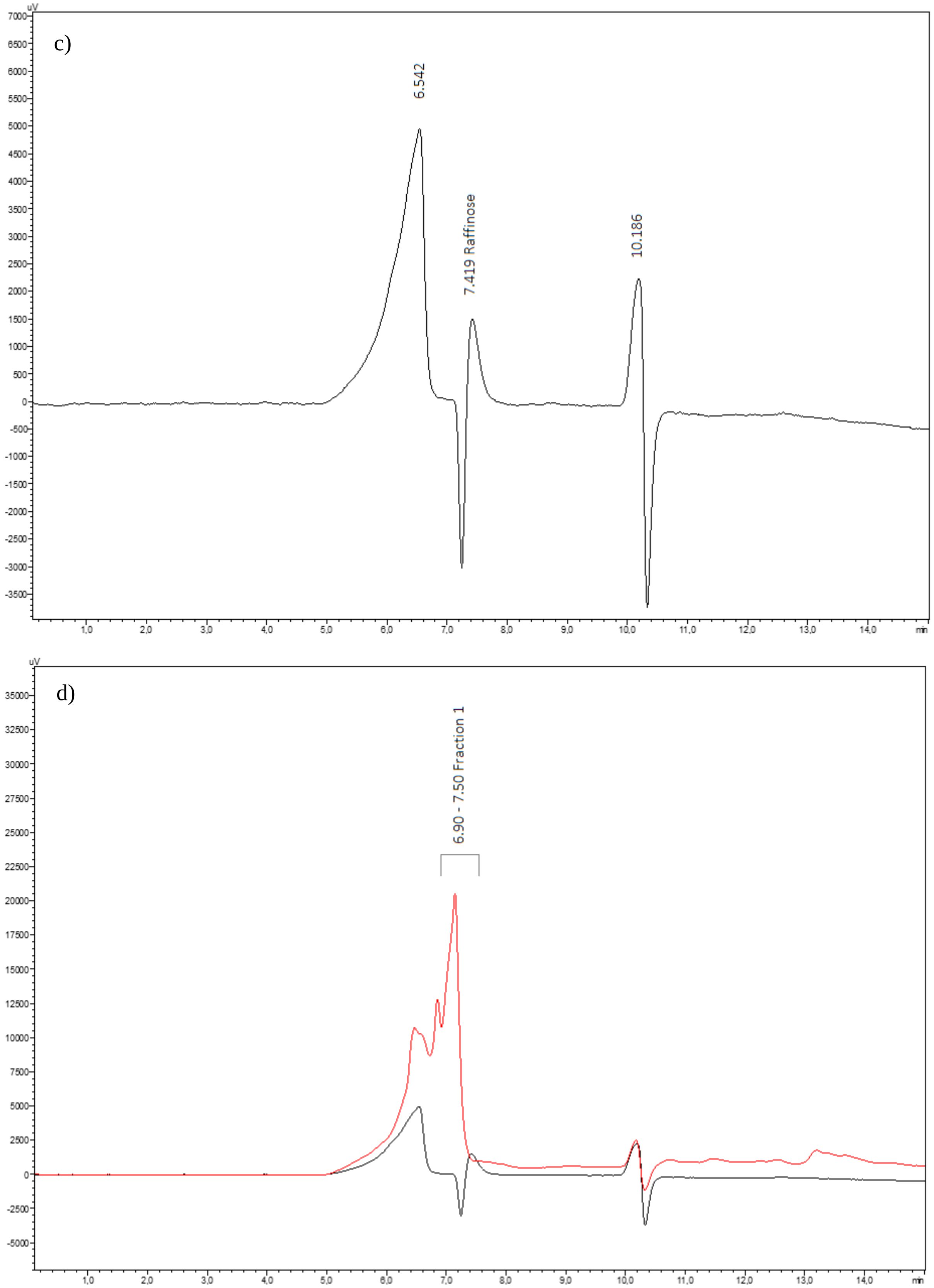

Figure S5. Preparative-HPLC chromatogram of a) willow hybrid Tora bark extract; b) overlay between the bark extract (red), raffinose (black) and a standard mixture (blue); c) authentic raffinose; d) overlay between Tora water extract (red) and raffinose (black) with marking of the separated raffinose-rich fraction.

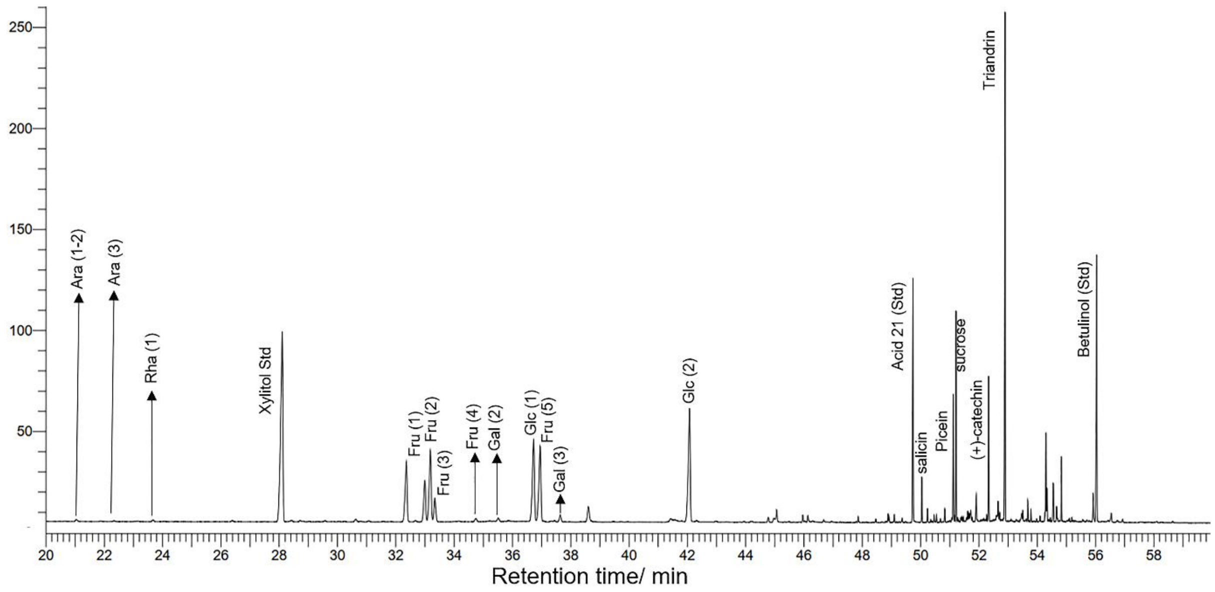

Figure S6. Gas chromatogram of trimethylsilylated Klara bark water extract with assignments of the major components. Temperature program I: Split: 1:30; Inj.: 250 °C; Column: 100 °C, 8 min, 2 °C/min, 170 °C, 12 °C/min, 310 °C for 5 min.

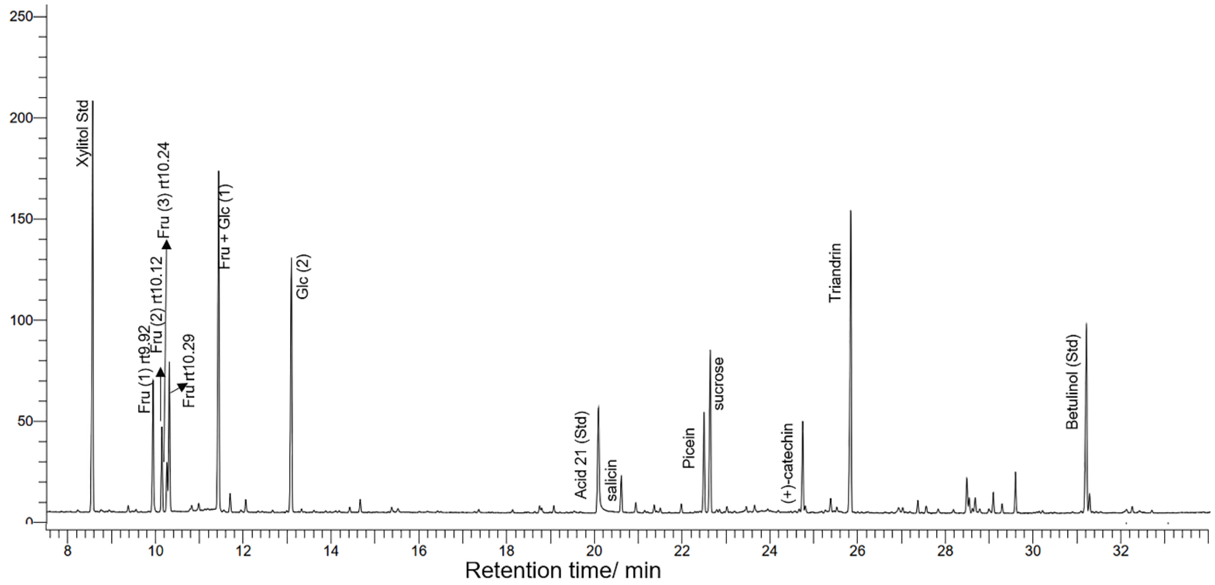

Figure S7. Gas chromatogram of trimethylsilylated Klara bark water extract with assignments of the major components. Temperature program II: Split: 1:25.8; Inj.: 250 °C; 120 °C, 1 min, 6 °C/min, 320 °C for 15min.

Table S3. Effect of particle size (cut to 2 cm or ground to 1mm) of willow bark (hybrids Klara and Karin) on gravimetric water extract yield. The extraction was carried out at 80 °C for 20 min.

	Yield (%)
Klara Bark (2cm)	14
Karin Bark (2cm)	12
Klara bark (1mm)	21
Karin bark (1mm)	16

Table S4. Effect of extraction temperature on the composition (mg/g) of water extract of bark of willow hybrid Klara based on one independent measurement. The extraction time was 20 min. The analytical techniques used for quantitative analyses were GC–FID with temperature program I (FID_M1) (Figure S6) and temperature program II (FID_M2) (Figure S7) and HPAEC–PAD (PAD). Abbreviations: Glucuronic acid (GlcA).

T (°C)	50			60			70			80			90			100		
Analytical technique	FID_M2	FID_M1	PAD	FID_M2	FID_M1	PAD	FID_M2	FID_M1	PAD	FID_M2	FID_M1	PAD	FID_M2	FID_M1	PAD	FID_M2	FID_M1	PAD
Salicin	5.1	4.0	—	5.1	4.0	—	5.1	4.0	—	8.0	6.0	—	7.1	5.7	—	8.3	6.4	—
Picein	22.2	19.5	—	22.1	19.3	—	22.1	19.0	—	22.2	17.5	—	21.7	19.0	—	21.1	18.3	—
(+)-Catechin	22.3	20.8	—	23.2	21.8	—	23.4	22.3	—	20.2	18.3	—	23.2	21.8	—	23.9	21.4	—
Triandrin	95.6	86.1	—	95.4	86.8	—	94.6	86.8	—	76.7	68.3	—	93.0	84.8	—	90.3	82.4	—
Total aromatic	145.2	130.4	—	145.8	131.9	—	145.2	132.2	—	127.4	110.1	—	145.0	131.3	—	143.6	128.4	—
Sucrose	17.7	10.8	—	14.6	10.5	—	14.7	9.2	—	32.1	22.2	—	16.3	11.1	—	14.1	10.2	—
Monosugars	142.7	152.2	187	150.6	162.5	146	147.2	155.7	141	159.5	174.4	169	133.4	141.8	155	118.1	130.3	134
Total sugars	160.4	163.0	—	165.2	173.0	—	161.9	164.8	—	191.6	196.6	—	149.7	152.9	—	132.2	140.5	—
GlcA	—	0.2	—	—	0.2	—	—	0.2	—	—	0.2	—	—	0.2	—	—	0.2	—

Table S5. Effect of extraction time on the composition (mg/g) of water extract of bark of willow hybrid Klara based on one independent measurement. The extraction temperature was 80 °C. The analytical techniques used for quantitative analyses were GC–FID with temperature program I (FID_M1) (**Figure S6**) and temperature program II (FID_M2) (**Figure S7**) and HPAEC–PAD (PAD). Abbreviations: Glucuronic acid (GlcA).

Time (min)	10			20			30			60			90			120		
Analytical technique	FID_M2	FID_M1	PAD	FID_M2	FID_M1	PAD	FID_M2	FID_M1	PAD	FID_M2	FID_M1	PAD	FID_M2	FID_M1	PAD	FID_M2	FID_M1	PAD
Salicin	5.4	4.3	—	8.0	6.0	—	5.8	4.8	—	6.3	4.9	—	7.7	6.0	—	7.8	—	—
Picein	21.7	18.0	—	22.2	17.5	—	20.1	17.8	—	21.2	18.5	—	21.3	17.8	—	21.8	—	—
(+)-Catechin	23.9	22.8	—	20.2	18.3	—	22.0	20.8	—	22.7	22.1	—	23.3	22.1	—	22.7	—	—
Triandrin	93.7	85.6	—	76.7	68.3	—	88.7	80.5	—	91.6	84.2	—	91.4	84.7	—	93.7	—	—
Total aromatic	144.8	130.7	—	127.4	110.1	—	136.7	124.0	—	141.9	129.6	—	143.6	130.6	—	145.9	—	—
Sucrose	22.1	11.3	—	32.1	22.2	—	19.5	17.7	—	20.6	16.4	—	16.0	9.9	—	15.7	—	—
Monosugars	128.3	138.6	149	159.5	174.4	169	119.0	144.2	151	116.1	131.6	145	122.1	131.7	138	125.4	—	152
Total sugars	150.4	149.9	—	191.6	196.6	—	138.5	161.9	—	136.7	148.0	—	138.1	141.6	—	141.1	—	—
GlcA	—	0.21	—	—	0.25	—	—	0.23	—	—	0.21	—	—	0.20	—	—	—	—

Table S6. Monosaccharide content (mg/g) of water extracts of willow (hybrid Klara) bark quantified by GC–FID (FID, temperature program I) and HPAEC–PAD (PAD) based on one independent measurement. Abbreviations: Arabinose (Ara), Rhamnose (Rha), Galactose (Gal), Glucose (Glc), Xylose (Xyl), Mannose (Man), Fructose (Fru). Abbreviation T80t10 refers to the extraction was performed for 10min under 80°C.

	T80t10		T80t20		T80t30		T80t60		T80t90		T50t20		T60t20		T70t20		T90t20		T100t20	
	FID-I	PAD	FID-I	PAD	FID-I	PAD	FID-I	PAD	FID-I	PAD	FID-I	PAD	FID-I	PAD	FID-I	PAD	FID-I	PAD	FID-I	PAD
Ara	0.4	0.9	0.4	0.6	0.2	0.8	0.2	0.8	0.3	0.8	0.4	1.1	0.4	0.9	0.2	0.7	0.4	0.9	0.3	0.8
Rha	0.0	0.8	0.6	0.8	0.5	0.7	0.5	0.6	0.1	0.6	0.0	1.0	0.6	0.8	0.6	0.7	0.0	0.6	0.5	0.7
Gal	3.2	3.9	3.3	3.6	3.3	3.9	3.0	3.7	2.7	3.5	3.3	4.7	3.5	3.8	3.4	3.6	3.0	4.0	2.7	3.6
Glc	49.3	68.6	67.0	82.1	53.5	68.9	47.4	66.6	48.8	63.6	54.1	87.0	58.6	66.8	56.1	65.6	51.4	70.7	46.9	64.5
Xyl	0.0	0.0	0.0	0.0	0.0	0.0	0.0	0.0	0.0	0.0	0.0	0.0	0.0	0.0	0.0	0.0	0.0	0.0	0.0	0.0
Man	0.1	0.2	0.4	0.5	0.0	0.2	0.0	0.2	0.1	0.1	0.0	0.1	0.0	0.1	0.0	0.2	0.0	0.1	0.0	0.3
Fru	85.7	74.9	102.7	81.0	86.7	76.4	80.6	73.2	79.7	69.8	94.3	92.8	99.4	73.9	95.3	70.6	87.0	78.8	79.9	70.0
monosaccharides	138.6	149.2	174.4	168.6	144.2	150.9	131.6	145.1	131.7	138.4	152.2	186.7	162.5	146.3	155.7	141.3	141.8	155.0	130.3	139.9

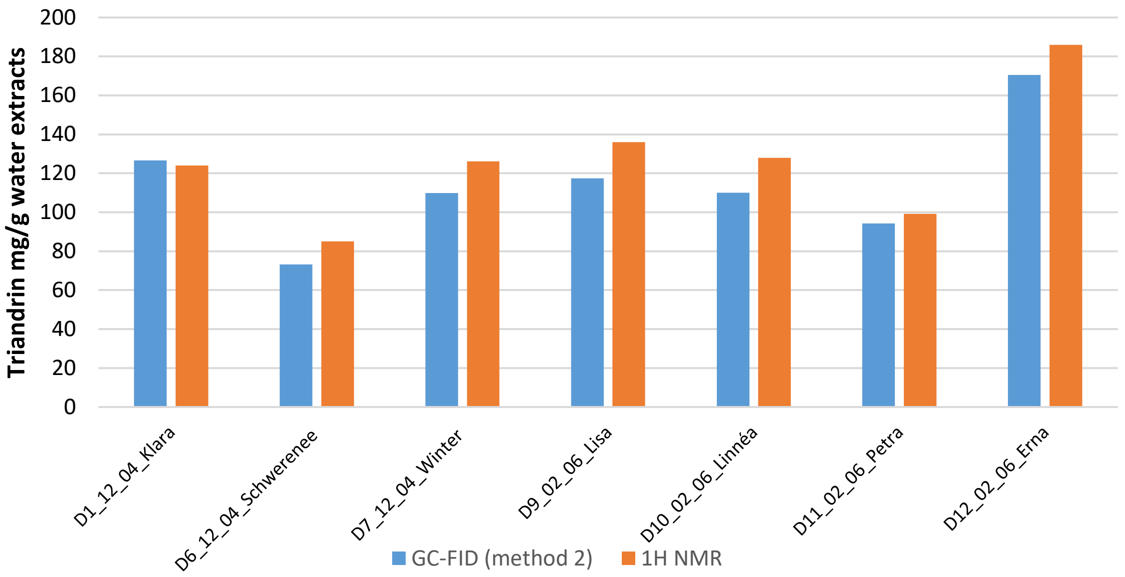

Figure S8. Content of triandrin in water extracts of seven willow hybrids (**Figure 5** and **Table S2**) analyzed by GC-FID (temperature program II, **Figure S7**) and ^{1}H NMR spectroscopy (integration of H-2 and H-3 protons relative to 1,3,5-trioxane) based on one independent measurement.

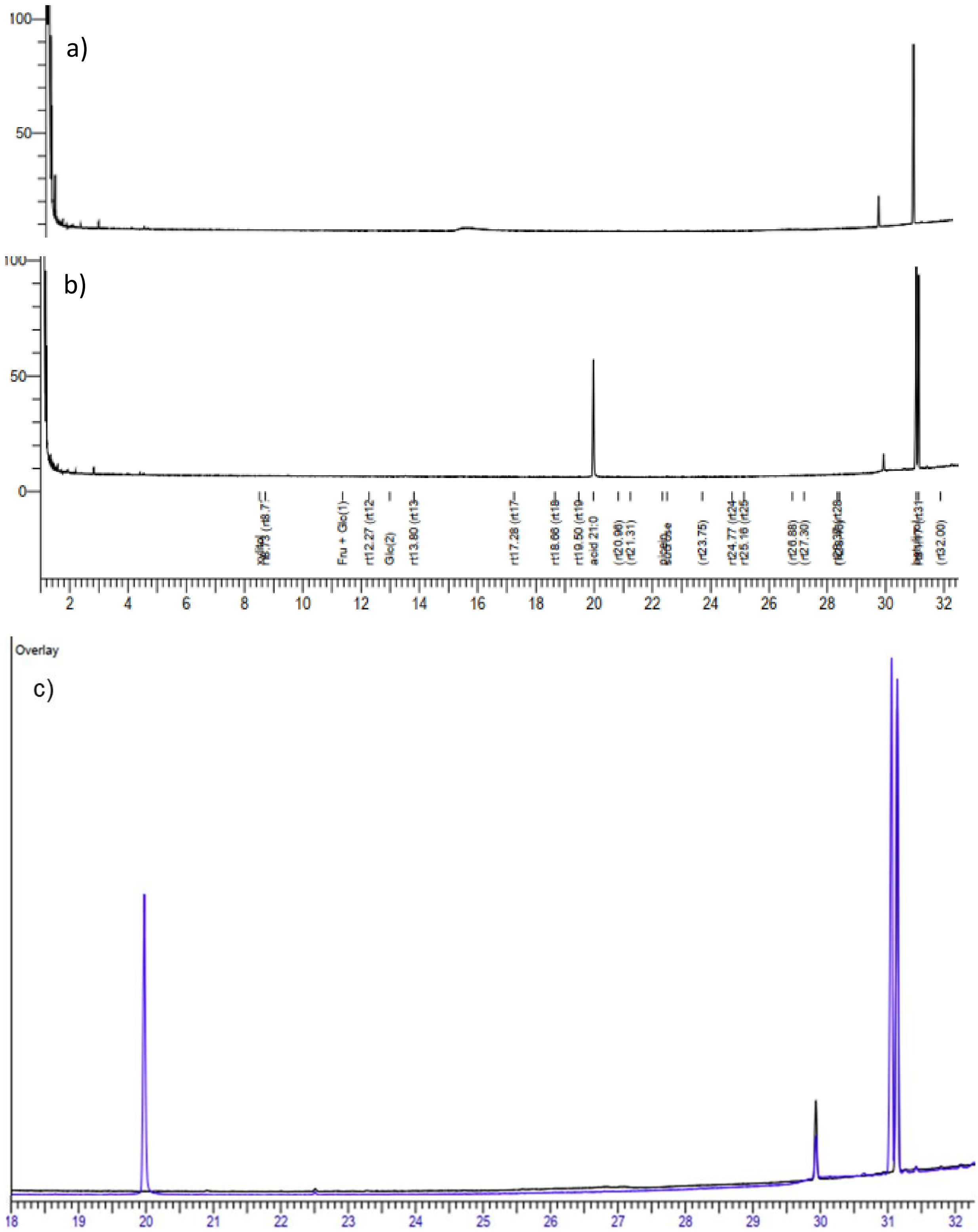

Figure S9. Gas chromatogram (*temperature program II*, **Figure S7**) of the trimethylsilyl derivatives of a) authentic raffinose; b) raffinose with added betulinol; c) overlay between chromatograms a and b.

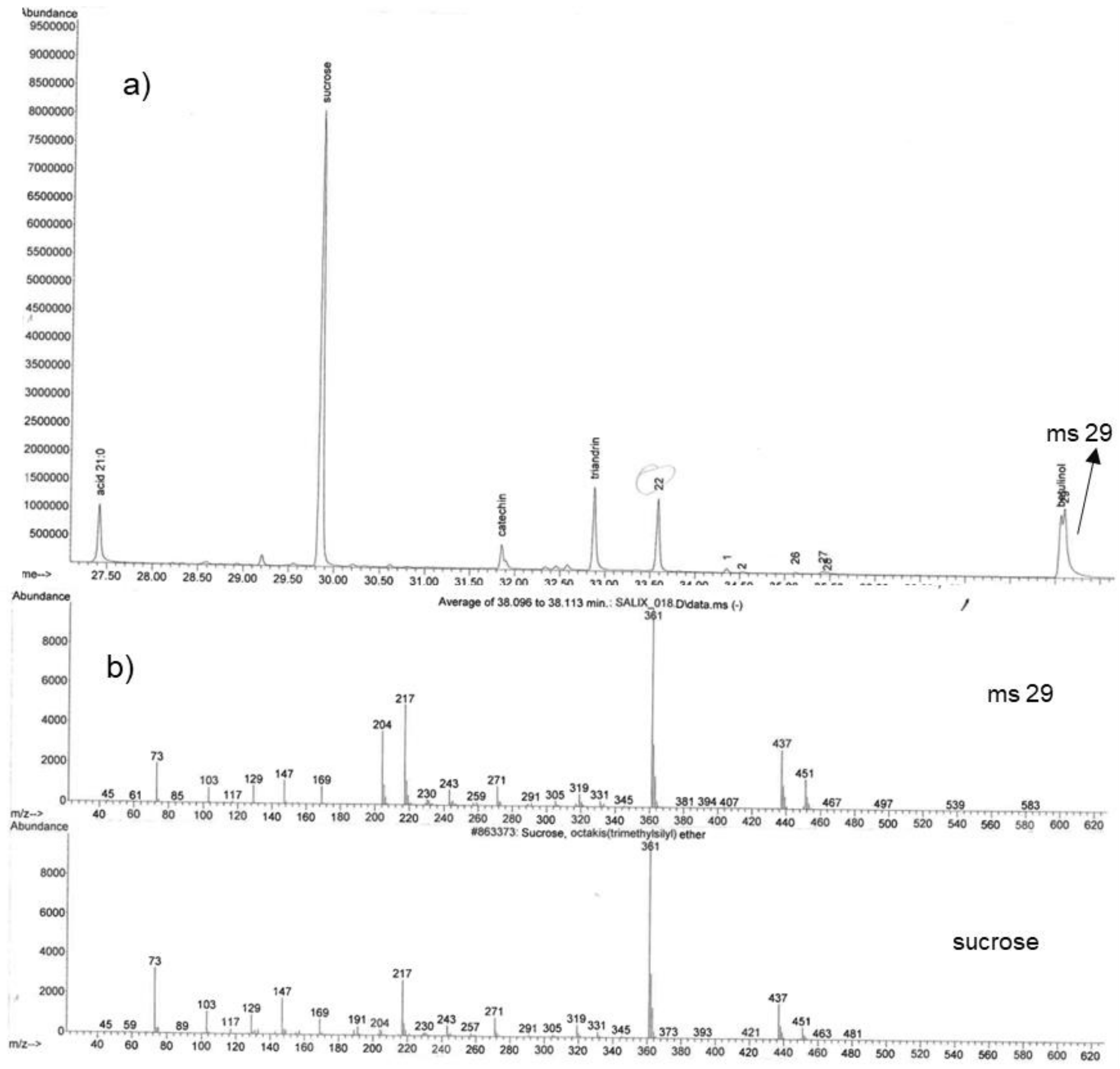

Figure S10. a) Gas chromatogram of trimethylsilylated willow hybrid Tora bark extract; b) mass spectra of peak ms 29 (raffinose) and sucrose. The GC-MS instrument was Hewlett Packard (HP) G1530A GC coupled to a HP MSD 5973. The column was Agilent Technologies HP-1, 25 m x 0.200 mm i.d., film thickness 0.11 µm. The carrier gas was He at flow rate of 0.8 mL/min. Program: Splitless, Inj.: 300 °C; Column: 80 °C, 1 min, 6 °C/min, 320 °C for 5 min.

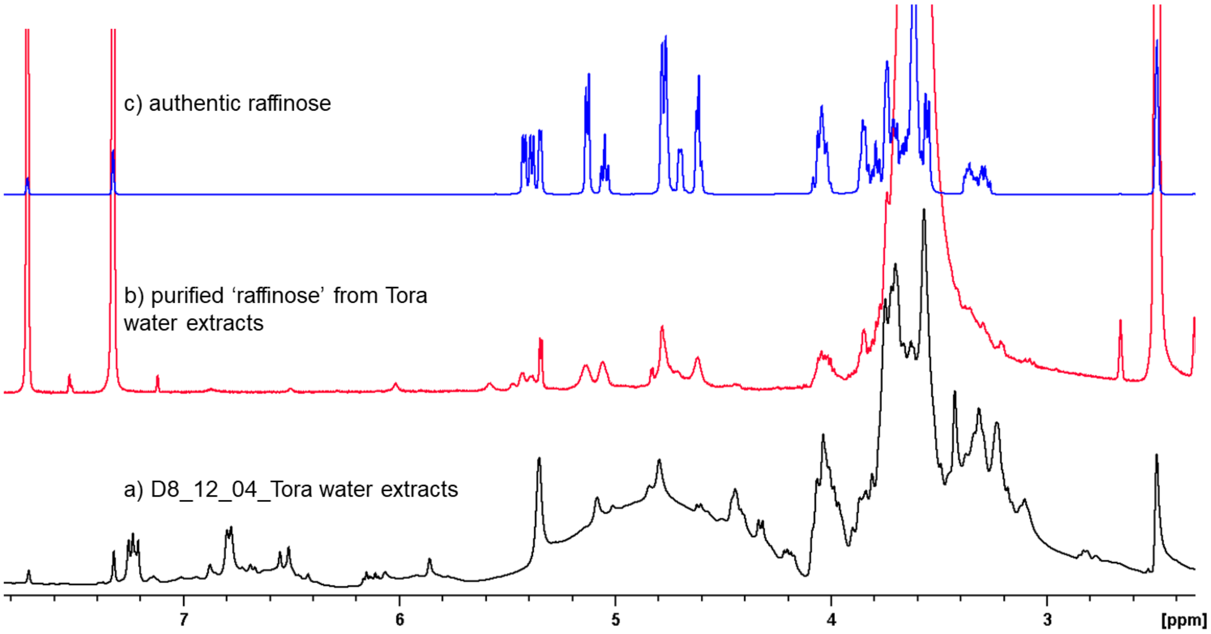

Figure S11. ^{1}H NMR spectra of a) Tora bark water extract; b) purified ‘raffinose-rich’ fraction; c) authentic raffinose in DMSO-*d*6/pyridine-*d*5.

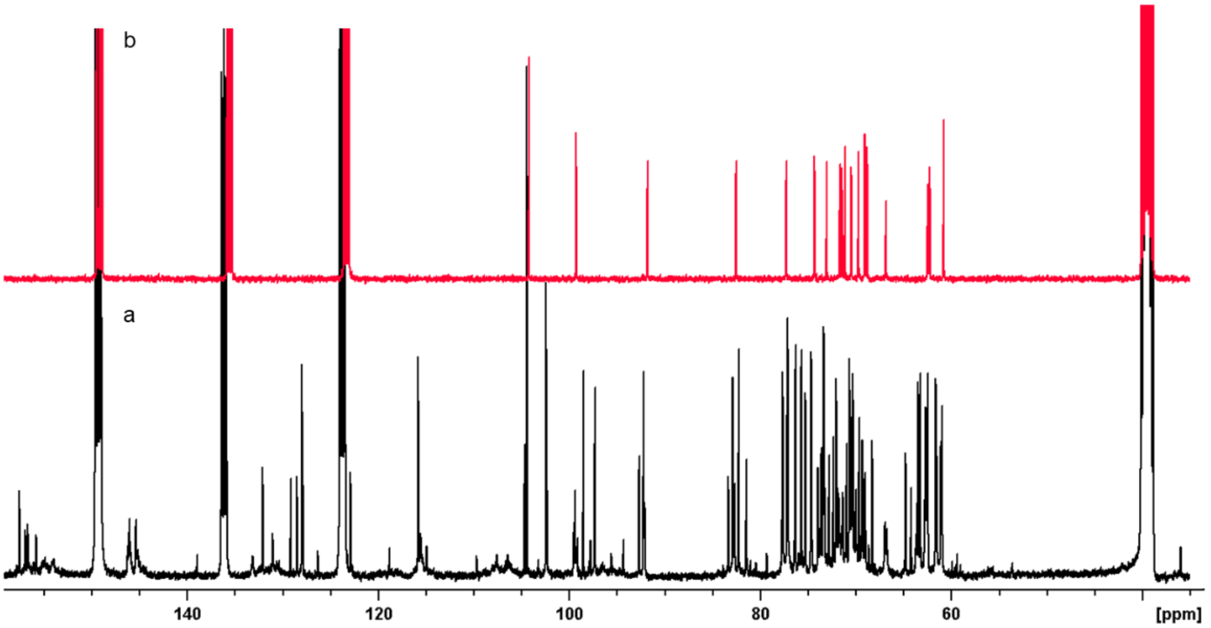

Figure S12. ^{13}C NMR spectra of a) Tora bark water extract; b) authentic raffinose in DMSO-*d*6/pyridine-*d*5. ^{13}C NMR spectrum of the purified ‘raffinose-rich’ fraction is not included as its concentration was low to get a proper spectrum.

Table S7. Standard deviations (included in the parentheses) for **Figure 4**. For abbreviations, see **Figure 4**.

Hybrid	content (mg/g extract)	02_17	04_24	07_12	09_23	12_04
Tordis	salicin	0.3 (0)	0.4 (0.1)	0.4 (0)	0.6 (0)	0.4 (0.1)
	picein	0.0 (0)	0.1 (0)	0.0 (0)	1.6 (0)	0.0 (0)
	(+)-catechin	12.7 (0.4)	10.4 (0.3)	7.3 (0.2)	26.4 (0.4)	16.7 (0.7)
	triandrin	37.0 (1.4)	42.3 (0.6)	38.2 (1.2)	59.3 (0.9)	50.2 (2.7)
	raffinose	16.3 (0.5)	2.4 (0)	0.7 (0)	11.5 (0.2)	66.8 (3.7)
	glucose + fructose	137.3 (0.4)	164.5 (4.2)	198.1 (9.7)	102.1 (1.2)	106.0 (2.3)
	sucrose	67.9 (1.4)	15.6 (0.4)	21.0 (2.0)	108.5 (2.3)	93.0 (5.2)
S. schwerenee	salicin	10.4 (0.1)	12.2 (0.2)	11.4 (0.1)	16.6 (0.1)	10.7 (0.2)
	picein	0.1 (0)	0.1 (0)	0.0 (0)	0.1 (0)	0.0 (0)
	(+)-catechin	11.0 (0)	10.5 (0.2)	7.1 (0)	22.5 (0.1)	15.0 (0.6)
	triandrin	48.8 (0.1)	60.8 (0.9)	70.4 (0)	143.0 (2.0)	73.2 (4.6)
	raffinose	16.6 (0.1)	1.2 (0)	0.3 (0)	12.7 (0.1)	83.1 (3.9)
	glucose + fructose	216.1 (4.6)	178.1 (15)	188.2 (1.7)	44.6 (1.0)	143.8 (2.6)
	sucrose	15.8 (0.6)	3.8 (0.5)	6.2 (0.1)	70.3 (3.4)	74.7 (3.5)
Winter	salicin	0.5 (0)	11.2 (0.2)	8.4 (0.3)	0.9 (0)	6.0 (0.2)
	picein	0.8 (0.1)	0.0 (0)	0.0 (0)	1.6 (0)	0.1 (0)
	(+)-catechin	14.7 (0.4)	18.5 (0.2)	9.5 (0)	26.5 (0.4)	19.6 (0.1)
	triandrin	29.9 (0.9)	145.2 (2.1)	98.0 (0)	93.0 (1.8)	109.9 (0.6)
	raffinose	28.7 (0.4)	1.9 (0)	0.5 (0)	8.0 (0.1)	119.2 (0.1)
	glucose + fructose	209.5 (5.0)	144.8 (3.9)	178.1 (0)	83.8 (2.0)	125.8 (4.3)
	sucrose	77.7 (0.3)	6.5 (0.2)	7.4 (0.2)	67.2 (2.8)	87.7 (5.7)
Tora	salicin	4.5 (0.1)	1.1 (0)	0.8 (0.1)	1.1 (0.2)	0.9 (0)
	picein	11.5 (0.5)	1.0 (0)	1.2 (0)	0.1 (0)	0.9 (0.1)
	(+)-catechin	19.3 (0.4)	10.6 (0.2)	10.1 (0)	18.1 (0.1)	18.8 (0.2)
	triandrin	49.7 (0.9)	32.8 (0.9)	36.2 (0.1)	101.5 (0.1)	39.1 (1.8)
	raffinose	85.8 (1.6)	1.1 (0.1)	0.4 (0)	23.0 (0.2)	103.1 (0.7)
	glucose + fructose	146.7 (4.9)	243.9 (18)	223.9 (3.8)	40.5 (1.1)	142.8 (0.9)
	sucrose	54.2 (2.8)	6.4 (0.5)	10.3 (0.1)	130.6 (7.8)	126.5 (6.3)

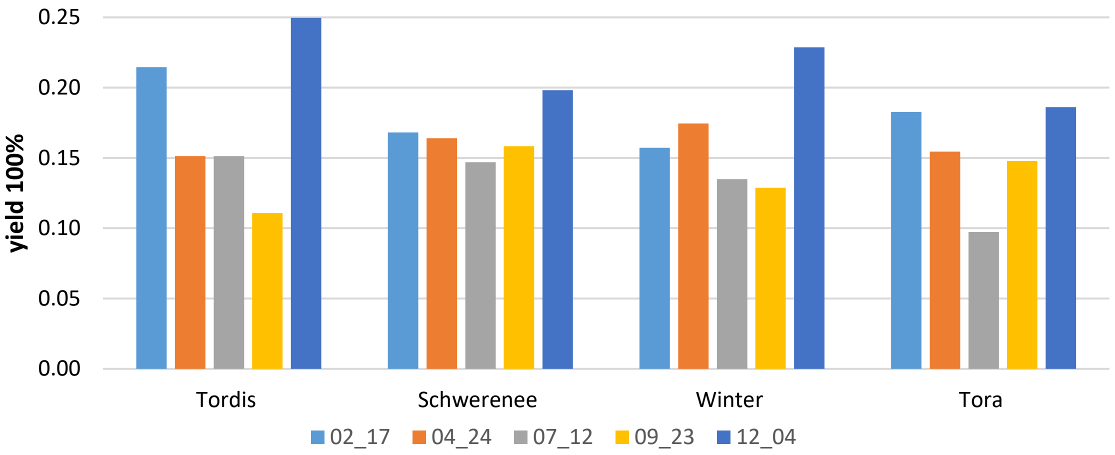

Figure S13. Hot water extract yield from selected *Salix* hybrids (Tordis, Schwerenee, Winter and Tora) based on one independent measurement. Sample abbreviations see **Figure 4**.

Table S8. Chemical composition (mg/g) of bark extracts from *Salix* hybrids quantified by gas chromatography (GC)-flame ionization detection (FID). For title abbreviations, see **Table S1**. SD = standard deviation.

	salicin (SD)	picein (SD)	(+)-catechin (SD)	(-)-epicatechin (SD)	triandrin (SD)	raffinose (SD)	glucose + fructose (SD)	sucrose (SD)	total assigned
D1_12_04_Klara	7.5 (0)	0.1 (0.1)	21.2 (0)	0.1 (0)	126.6 (0.3)	30.6 (0.5)	148.6 (0.1)	45.5 (0.6)	380
D2_10_17 Karin	1.3 (0.2)	50.7 (1.8)	19.2 (0.6)	0.2 (0.1)	44.9 (1.3)	0.5 (0.1)	143.7 (2.4)	1.1 (0.9)	262
D3_10_17_myrsinifolia	23.7 (0.8)	114.4 (7.0)	12.4 (0.7)	0.2 (0)	8.3 (0.5)	0.6 (0.1)	124.6 (0.1)	0.8 (0.6)	285
D4_10_17_schweinni	0.6 (0.1)	0.2 (0)	13.2 (0)	0.4 (0.1)	48.1 (0.7)	0.7 (0)	117.8 (0)	1.1 (0.6)	182
D5_12_04_Tordis	0.4 (0.1)	0.0 (0)	16.7 (0.7)	0.1 (0.1)	50.2 (2.7)	66.8 (3.7)	106.0 (0.3)	93.0 (0.5)	333
D6_12_04_Schwerenee	10.7 (0.2)	0.0 (0.1)	15.0 (0.6)	0.0 (0)	73.2 (4.6)	83.1 (3.9)	143.8 (0.8)	74.7 (0.9)	400
D7_12_04_Winter	6.0 (0.2)	0.1 (0)	19.6 (0.1)	0.0 (0)	109.9 (0.6)	119.2 (0.1)	125.8 (0.2)	87.7 (0.4)	468
D8_12_04_Tora	0.9 (0)	0.9 (0.1)	18.8 (0.2)	0.0 (0)	39.1 (1.8)	103.1 (0.7)	142.8 (0.8)	126.5 (0.8)	432
D9_02_06_Lisa	0.0 (0)	0.0 (0)	18.2 (0.1)	0.2 (0)	117.4 (1.1)	27.5 (0.3)	204.2 (0.2)	16.4 (0.7)	384
D10_02_06_Linnéa	7.5 (0.1)	2.0 (0)	21.1 (0)	0.1 (0.1)	110.1 (0.4)	17.0 (0.3)	167.2 (0.2)	16.3 (0)	341
D11_02_06_Petra	8.3 (0.6)	0.2 (0)	25.3 (0.2)	0.0 (0)	94.2 (0.9)	27.6 (1.4)	128.1 (0.2)	78.8 (0.6)	363
D12_02_06_Erna	19.1 (0.3)	3.9 (0.1)	19.8 (0.5)	0.0 (0)	170.6 (4.1)	19.5 (0.7)	127.5 (0.2)	52.4 (0.1)	413
D13_02_06_Gertrud	0.4 (0)	0.3 (0)	22.3 (0.4)	0.1 (0)	136.3 (0.6)	8.6 (0.1)	151.7 (0.1)	15.8 (0.5)	336
D14_02_06_Julia	14.3 (0)	2.4 (0.1)	14.4 (0)	0.0 (0)	61.1 (0.3)	24.0 (0.4)	143.0 (0.1)	28.0 (0.2)	287
015_09_23_Agnieska	0.6 (0)	0.2 (0.1)	25.6 (0.1)	0.6 (0.3)	107.2 (0.2)	20.3 (0.2)	25.8 (0)	104.1 (2.2)	284

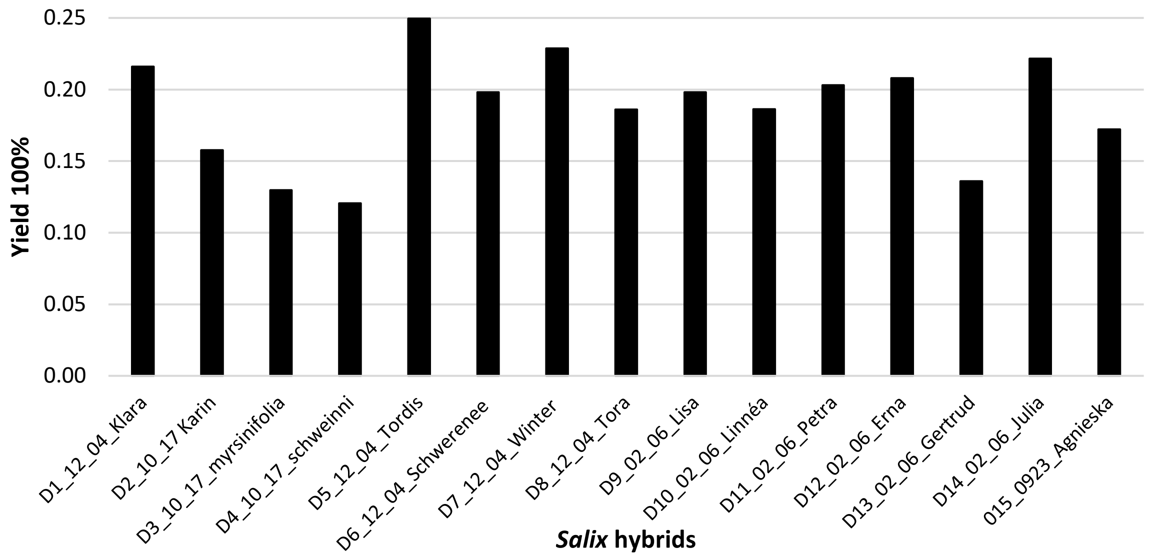

Figure S14. Hot water extract (HWE) yield from *Salix* hybrids based on one independent measurement. Title abbreviation key: code_harvesting month_day_hybrid name.

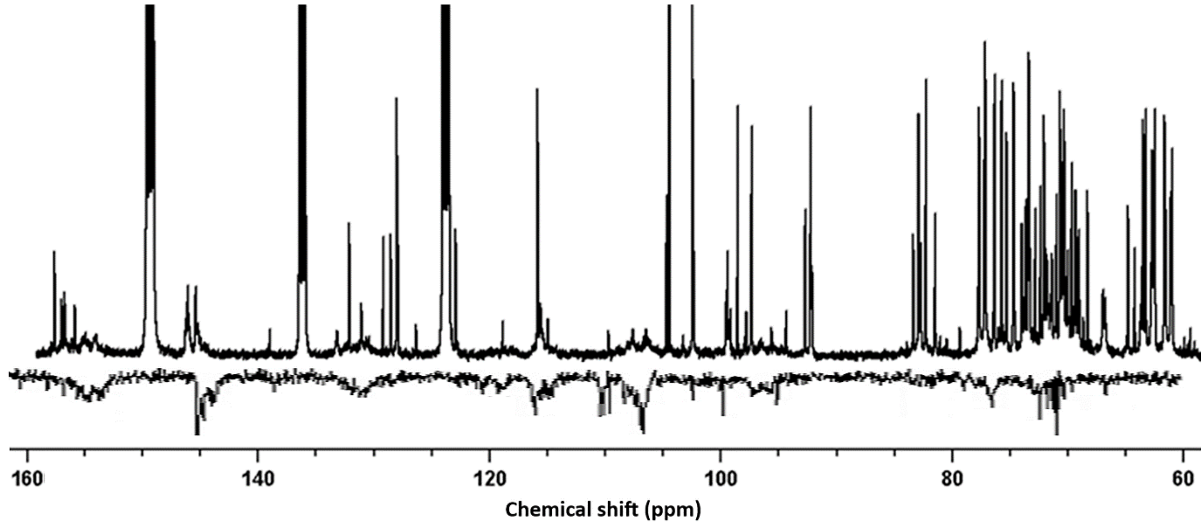

Figure S15. Comparison between ^{13}C NMR spectra of Tora bark extract in DMSO-*d*6:pyridine-*d*5 (4:1) (top) and condensed tannin from leaves of *Leucaena leucocephala* hybrid Rendang in D_2O/acetone-d6 (1:1) (down, inverted)[2].

Table S9. Abundance of the bioactive component from each selected hybrids and screening of willow hybrid water extracts (1 mg/mL) against four bacterial strains. The inhibitory effect was assessed visually. “++” refers to no detectable bacterial growth in the wells, “+” indicates partial growth inhibition, and “-“ no bacterial growth inhibition.

Hybrid	Abundant bioactive component	Bacterial strain			
		Escherichia coli ATCC 25922	*Staphylococcus aureus* ATCC 29213	*Enterococcus faecalis* ATCC 29213	*Salmonella typhimurium* ATCC 19585
**Klara**	triandrin	-	++	-	-
**Karin**	picein; triandrin	-	++	-	-
S. myrsinofolia	picein	-	++	-	-
**Tora**	raffinose	-	++	-	-
**Erna**	triandrin	-	++	-	-

Table S10. Raw data for acute test to *Daphnia similis* performed for 48 h at 21 °C.

Concentration (mg/L)	Number of immobilized organisms				Total	%
	1	2	3	4		
0	0/5	0/5	0/5	0/5	0/20	0
DMSO control (0.1%)	0/5	0/5	0/5	1/5	1/20	5
0.1	0/5	0/5	0/5	0/5	0/20	0
0.25	0/5	0/5	0/5	0/5	0/20	0
1	0/5	0/5	0/5	0/5	0/20	0
2.5	0/5	0/5	0/5	0/5	0/20	0
10	0/5	0/5	0/5	0/5	0/20	0
25	0/5	0/5	0/5	0/5	0/20	0

Reference

[1] Dou, J.; Xu, W.; Koivisto, J. J.; Mobley, J. K.; Padmakshan, D.; Kögler, M.; Xu, C.; Willför, S.; Ralph, J.; Vuorinen, T. Characteristics of Hot Water Extracts from the Bark of Cultivated Willow (*Salix* sp.). *ACS Sustainable Chem. Eng.* **2018**, *6*, 5566–5573.

[2] Zarin, M. A.; Wan, H. Y.; Isha, A.; Armania, N. Antioxidant, antimicrobial and cytotoxic potential of condensed tannins from *Leucaena leucocephala* hybrid-Rendang. *Food Sci. Hum. Wellness* **2016**, *5*, 65–75.
